# A novel small molecule glycolysis inhibitor WZ35 exerts anti-cancer effect via metabolic reprogramming

**DOI:** 10.1186/s12967-022-03758-0

**Published:** 2022-11-18

**Authors:** Lihua Wang, Zheng Zhu, Qi Liang, Yecheng Tao, Gaowei Jin, Yaoyao Zhong, Jichen Dai, Ruixia Dai, Zhixiang Wang, Junbo Chen, Lingjie Zhou, Shouyu Ke, Bin Zheng, Linhua Lan, Xiaokun Lin, Tongke Chen

**Affiliations:** 1 School of Ophthalmology and Optometry, Eye Hospital, Wenzhou Medical University, Wenzhou, 325000 Zhejiang China; 2 State Key Laboratory of Ophthalmology, Optometry and Visual Science, Wenzhou, 325000 Zhejiang China; 3 Department of Endocrine and Metabolic Diseases, Ruijin Hospital, Shanghai Jiao Tong University School of Medicine, Shanghai, 200025 China; 4 Laboratory Animal Centre, Wenzhou Medical University, Wenzhou, 310020 Zhejiang China; 5 Department of Cardiology, Sir Run Run Shaw Hospital, College of Medicine, Zhejiang University, Hangzhou, 310000 Zhejiang China; 6 Key Laboratory of Diagnosis and Treatment of Severe Hepato-Pancreatic Diseases of Zhejiang Province, The First Affiliated Hospital of Wenzhou Medical University, Wenzhou, 325000 Zhejiang China; 7 Department of Pediatric Surgery, The Second Affiliated Hospital and Yuying Children’s Hospital, Wenzhou Medical University, Wenzhou, 325027 Zhejiang China

**Keywords:** Hepatocellular carcinoma, WZ35, Metabolic reprogramming, GLUT1, YAP

## Abstract

**Background:**

Liver cancer is the fifth leading cause of cancer death worldwide, but early diagnosis and treatment of liver cancer remains a clinical challenge. How to screen and diagnose liver cancer early and prolong the survival rate is still the focus of researchers.

**Methods:**

Cell experiments were used to detect the effect of WZ35 on the colony formation ability and proliferation activity of hepatoma cells, nude mouse experiment to observe the in vivo anticancer activity and toxic side effects of WZ35; metabolomics analysis, glucose metabolism experiment and Seahorse analysis of liver cancer cells treated with WZ35; cell experiments combined with bioinformatics analysis to explore the mechanism of WZ35-mediated metabolic reprogramming to exert anticancer activity; tissue microarray and case analysis to evaluate the clinical significance of biomarkers for early diagnosis, treatment and prognosis evaluation of liver cancer.

**Results:**

WZ35 inhibited the proliferation activity of various cell lines of liver cancer, and showed good therapeutic effect in nude mice model of hepatocellular carcinoma without obvious toxic and side effects; WZ35 inhibited the absorption of glucose in hepatoma cells, and the drug effect glycolysis, phosphorylation and purine metabolism are relatively seriously damaged; WZ35 mainly inhibits YAP from entering the nucleus as a transcription factor activator by activating oxidative stress in liver cancer cells, reducing the transcription of GLUT1, and finally reducing its GLUT1. Tissue microarray and case analysis showed that GLUT1 and YAP were highly expressed and correlated in liver cancer patients, and were associated with poor patient prognosis. The GLUT1-YAP risk model had a high score in predicting prognosis.

**Conclusion:**

The study confirms that WZ35 is a small molecule glycolysis inhibitor, and through its properties, it mediates metabolic reprogramming dominated by impaired glycolysis, oxidative phosphorylation and purine metabolism to inhibit the proliferation activity of liver cancer cells. Our findings present novel insights into the pathology of liver cancer and potential targets for new therapeutic strategies. GLUT1-YAP has important reference significance for predicting the stages of disease progression in liver cancer patients and have the potential to serve as novel biomarkers for the diagnosis and treatment of liver cancer.

**Supplementary Information:**

The online version contains supplementary material available at 10.1186/s12967-022-03758-0.

## Introduction

Hepatocellular carcinoma is the fifth most common cancer worldwide and the second leading cause of cancer-related death for men worldwide, with 905,677 new cases and 830,180 new deaths of liver cancer in 2020. In China, despite the decline in the prevalence of HBV and HCV, the increase of excess body weight and diabetes have still stabilized the incidence rates of hepatocellular carcinoma at a high level [1].

Cells undergo metabolic reprogramming during carcinogenesis, including dysregulation of glycolysis, glutamine addiction, reprogramming of lipid metabolism, and more. Targeting cancer metabolism is believed to have promising anticancer effects [2]. High demand for acquiring metabolism resources lead to fierce nutrient competition in the tumor microenvironment. With greater capacity to consumpt glucose, tumor cells can build up a glucose-depleted environment to restrict T cells function, inducing their dampened mTOR activity, glycolytic capacity, and IFN-g production. T cell dysfunction or hyporesponsiveness allow tumor immune escape [3]. Ketogenic diet or exogenous supplements can induce ketosis, significantly reduce blood glucose and insulin, modulate tumor microenvironment, as well as slow down tumor progression and potential symptom burden [4].

Glucose, as the most important energy source of the cell, plays an extremely important role in the progression of the tumor cells [5]. Blood glucose level could be directly correlated with tumor growth [6]. ‘ Warburg effect’ refers to an important change in the glucose metabolism of tumor cells [7]. Glycolysis of tumor cells is active even under aerobic conditions, showing high glucose uptake rate, high lactic acid content of the metabolites, and high ratio of glycolysis of the cancer cells to meet the various metabolic needs of the cells. It requires a large amount of the glucose transport from the tissue fluid into the cell, which is mainly mediated through the different glucose transporters (GLUTs). At present, there are 14 members belonging to this family, among which GLUT1 is the most important [8], with two domains constitute a typical inward conformation [9]. Since tumor cells need to express a great quantity of GLUT1 to meet their huge energy needs, GLUT1 expression in a variety of tumors is increased, such as that of lung cancer, hepatocellular carcinoma, gastric cancer and colorectal cancer [10–13]. Kurahara et al. found that the prognosis of patients with pancreatic ductal adenocarcinoma was directly related to the expression of GLUT1 [14]. Because of the importance of GLUT1 in the regulation of metabolism of tumor cells, drug targeting GLUT1 to regulate the metabolism could be prominent in cancer therapy.

YAP, also known as YAP1, is a transcription regulator of the Hippo pathway and hasimportant biological properties in cell proliferation, mature cells dedifferentiation, tumorigenesis and reprogramming of cellular metabolism [15]. When the pathway is activated, YAP accumulates in the nucleus and the transcriptional co-activators of YAP and TAZ are recruited to target site by TEAD. YAP/TAZ/TEAD and activator protein-1 form a complex which promotes the expression of target genes [16]. Another recent study has also proposed a new model in which YAP/TAZ is recruited to target genomic regions by a complex of AP-1, STAT3, and TEAD proteins [17]. The overexpression of YAP in the liver of transgenic animals can induce the proliferation of mature hepatocytes and ultimately lead to a four-fold increase in liver mass [18]. Its overexpression can also cause hyperproliferation of and depressed differentiation of neural progenitor cells [19]. YAP has been detected in late-stage ovarian, colon, gastric, liver, esophageal and lobular type of invasive breast cancers to foster their proliferation, progression and migration [20]. In recent years, studies have found that YAP is an emerging node that coordinates cell nutrition and tissue homeostasis, and participates in the regulation of cell metabolism such as glycolysis, lipogenesis, and glutaminolysis [21, 22].

WZ35 was synthesized by our team [23], which can significantly affect the growth of gastric cancer [24], hepatocellular carcinoma [25] and breast cancer [26]. In this article, we further found that WZ35 could negatively regulate the metabolism of liver cancer cells mediated through GLUT1. The relationship between YAP and GLUT1 remains unclear. Andrew G Cox et al. have used zebrafish embryos as a model to prove YAP regulates the expression of GLUT1 and thus induces the regeneration of organs [27]. But the effect on the metabolism and regulation mechanism remains to be further explored.

In this report, we found a novel small molecule glycolysis inhibitor WZ35 that could inhibit the intake of glucose. We identified new role of GLUT1-YAP on the prognosis of liver cancer patients. Further experiments demonstrated glucose intake inhibition inducing metabolic reprogramming and thereby suppressing the proliferation of liver cancer cells.

## Material and methods

### Reagents

Curcumin obtained from Sigma (MO, USA). WZ35, a monocarbonyl curcumin analog, was prepared as described in prior studies [28]. Cell Counting Kit-8 (CCK-8) and horseradish peroxidase (HRP)-conjugated anti-rabbit and anti-mouse IgG and N-acetyl-l-cysteine (NAC) were purchased from Beyotime (Haimen, China). Cell Apoptosis DAPI Detection Kit, DCFH-DA ROS detection kit, and pEGFP-hYAP 1 were obtained from Addgene (Shanghai, China). A Hippo Signaling Antibody Sampler Kit (#8579), anti-YAP(#14074S), anti-Cleaved caspase 3 (#9661), anti-Bax (#2774), anti-Bcl-2 (#2870), anti-E-cadherin (#8834S), anti-GLUT1 (#73015), anti-N-cadherin (#13116S), anti- Histone H3 (#9715S) and anti-GAPDH (#D16H11) were purchased from Cell Signaling Technology (USA) for Western blot and IHC. Anti-Cyr61 (Sc-374129), anti-CTGF (Sc-365970) were purchased from Santa Cruz Biotechnology (USA) for Western blot. Anti-Tublin (ac008) was purchased from Abclonal for Western blot.

### Cell culture

The human liver cancer cell lines including HCCLM3, HepG2 and Huh7 were obtained from the Institute of Biochemistry and Cell Biology, Chinese Academy of Sciences. The cells were grown using Dulbecco's Modified Eagle's Medium (DMEM) containing 10% FBS (Life Technologies) and penicillin/streptomycin at 37 °C in a humidified 5% CO_2_ incubator.

### Patient samples

110 matched liver cancer samples and precancerous control tissues were isolated from untreated patients at the Department of Hepatopancreatobiliary Surgery of the First Affiliated Hospital of Wenzhou Medical University, Wenzhou, China, between 2010 and 2014. They were separated into two different portions, with one portion being used for routine histopathological examinations, and the rest being snap-frozen and stored at −80 °C for future studies. Liver cancer was pathologically confirmed in all patients, with tumors staging being determined based upon the AJCC/UICC (Union for International Cancer Control/American Joint Committee on Cancer) and WHO classification. The Board and Ethical Committee of the First Affiliated Hospital of Wenzhou Medical University approved this study (ID number: wydw2019-0446), and all the patients provided written informed consent.

### Bioinformatics analysis

The GENE EXPRESSION OMNIBUS (GEO) database was searched using the terms "gene expression", "hepatocellular carcinoma", and "Homo sapiens". We selected the GSE14520 dataset as it met the quality control and the sample size criteria. The R software (version.4.2.0) limma package was used for normalization. Based on quality filtering, two healthy control microarray samples were excluded, while the remaining 486 microarrays (22 liver cancer samples, 19 adjacent control samples based on the GPL571 platform and 225 liver cancer samples, 220 adjacent control samples based on the GPL3921 platform were retained for analysis. The RStudio Limma package was used to identify the differentially expressed genes (adjusted *P* < 0.05 and | logFC (Fold Change) |≥ 0.5), after which probe names were converted to gene names. The ggplot2 package was used to visualize differences in gene expression between the samples, with appropriate functions also being used to draw the boxplots, survival curves, and volcano plots in R software (version.4.2.0). To reveal the potential biological functions and underlying mechanisms of genes, we used the R package “clusterProfiler” to analyze Gene Ontology (GO) and Kyoto Encyclopedia of Genes and Genes (KEGG) term enrichment of the target genes GO terms, including biological processes (BPs), cellular components (CCs), molecular functions (MFs), and KEGG pathways with adjusted *P* < 0.05, were considered statistically significant. JASPAR [29] and USCS [30] were used to predicted binding sites, conservation and histone modification.

### Cell proliferation assay

Liver cancer cells were plated in 96-well plates (8 × 10^3^/well) overnight, after which they were untreated or treated with curcumin (10 μg/mL), WZ35 (10 μg/mL), NAC (5 mM). For some experiments, a range of drug concentrations (0, 5, 10, 20, and 40 μg/mL) were used instead. WZ35 and curcumin were prepared using 0.03% DMSO; NAC was prepared in PBS and diluted using complete media. The CCK-8 kit was used to measure the viability with absorbance at 450 nm being quantified via microplate reader (Spectramax m5, Molecular Devices).

### Colony formation assay

HCCLM3 cells were plated in 6-well plates (5,000/well) with curcumin (1 μg/mL), WZ35 (1 μg/mL), and/or NAC (5 mM). The plate incubated until individual colonies were formed containing approximately 50 cells. The cells were then washed in PBS, fixed for 20 min with methanol, stained for 15 min with crystal violet, washed with distilled water, and colonies were counted using the ImageJ software.

### DAPI staining for cells

Apoptosis was measured by plating HCCLM3 cells in 6-well plates overnight (5 × 10^5^/well). The cells were then treated using WZ35 or curcumin (both 10 cg/mL) for 18 h, after which they were washed using PBS, fixed for 15 min in 4% paraformaldehyde (PFA), and stained for 15 min with 4',6-diamidino-2-phenylindole (DAPI) (400 μL). The cells were thereafter imaged via microscope (NIKON, Japan) with 100 magnification. Compared to normal cells, cells undergoing apoptosis presented enhanced DAPI fluorescence for condensed nuclear chromatin, which can be individually identified with ImageJ software. Apoptosis rate was calculated by the percentage of condensation nuclear identified by ImageJ.

### Western blotting

Radio immunoprecipitation assay (RIPA) lysis buffer obtained from Beyotime (Haimen, China) was used to isolate the proteins from the cells or to homogenize the tumor tissues. The samples were then spun for 15 min at 12,000 rpm, and equal amounts of supernatant protein (10 μg/sample) were separated via sodium dodecyl sulfate–polyacrylamide gel electrophoresis (SDS-PAGE) (8, 10 or 12%) prior to transfer onto a poly (1,1-difluoroethylene) (PVDF) membrane (Millipore, Burlington, MA, USA). The blots were blocked for 90 min using 5% skim milk in TRIS hydrochloride & Tween (TBST), followed by an overnight incubation with the various primary antibodies at 4 °C. After washing three times in TBST (10 min/wash), the blots were incubated for 1 h with horseradish peroxidase (HRP)-linked secondary antibodies (1:2000), after which they were washed again and protein bands were detected using an electrochemiluminescence (ECL) reagent (Thermo Scientific, IL, USA). The samples were subjected to western blot analyses as described previously [25].

### Total quantitative real-time PCR analysis

RNAs were isolated from cells using Triozol reagent (sigma) according to the manufacturer’s instructions. The quantity and quality of the extracted total RNA were assessed by using a Nanodrop 1000 spectrophotometer (Thermo Scientific). One microgram of total RNA was reverse-transcribed into complementary DNA with HiScript III 1st Strand cDNA Synthesis Kit with gDNA Wiper (Vazyme;R312-01). Quantitative PCR was performed with SYBR qPCR Master Mix (Vazyme; MQ101-01) and quantified by the StepOne Real-Time PCR System (Applied Biosystems). The signals were detected and analyzed on CFX Connect System (Bio-rad, USA). GAPDH was used as an internal control. The amplification parameters were set at 95 ℃for 30 s, 60 ℃ for 30 s, and 95 ℃ for 10 s (40 cycles total). Gene-specific primers used in the study were:

YAP: TGTCCCAGATGAACGTCACAGC (forward), TGGTGGCTGTTTCACTGGAGCA(reverse);

GLUT1: TTGCAGGCTTCTCCAACTGGAC(forward), CAGAACCAGGAGCACAGTGAAG(reverse); GAPDH:GTCTCCTCTGACTTCAACAGCG(forward), ACCACCCTGTTGCTGTAGCCAA(reverse).

### Transmission electron microscopy (TEM)

Curcumin- and WZ35-induced changes in the cellular structure were visualized via TEM, with DMSO being used as a control. The treated cells were pre-fixed overnight in primary fixative (2.5% glutaraldehyde and 1 mM CaCl_2_ in 0.15 M cacodylate buffer), followed by a 2 h post-fix in 1% osmium tetroxide. Subsequently, specimens were dehydrated in an ascending ethanol gradient (50%, 70%, 90%, 95% and 100% three times; each step 5 min) and in isoamyl acetate 15 min. Ultrathin pellet sections were then stained using 2% aqueous uranyl acetate and lead citrate, after which a TEM instrument (Wenzhou Medical University, H-7500, HITACHI, Japan) was used to image the cells with 26,500 magnification.

### Oxidative phosphorylation and glycolysis measurements

A Seahorse XF96 Extracellular Flux Analyzer (Agilent Technologies, CA, USA) was used for oxygen consumption rate (OCR) and extracellular acidification rate (ECAR) measurements. 2 × 10^4^ cells add to machine-specific plates. Drug doses were used to treat cells for 6 h. Following probe calibration, OCR was measured via sequentially injecting oligomycin (ATP synthase inhibitor; 1 µM), FCCP (uncoupler; 0.5 µM), rotenone (complex I inhibitor; 1 µM), and antimycin A (complex III inhibitor; 2 µM). ECAR was measured using the cells treated in the same manner via injection of glucose (10 mM), oligomycin (1 µM), or 2-DG (100 mM).

### Flow cytometry analysis for ROS determination

ROS production was quantified by staining the cells for 30 min with 10 μM 2,7-dichlorofluo-rescein diacetate (DCFH-DA) in DMEM at 37 °C. The cells were then treated for 2 h with corresponding compounds (10 μg/mL), washed twice with DMEM, and DCFH-DA fluorescence was quantified by BD Accuri TM C6 flow cytometer (BD, Franklin Lakes, NJ).

### Detection of glucose in the supernatant

Cells were plated in 3 dishes (60 mm) with DMSO (10 μL/mL), WZ35 (10 μg/mL) and blank. After a 18 h-drug-treatment, the supernatant was collected. According to Beckman Kurt biochemical analysis system, glucose determination kit (Beckman Coulter, CA, USA) was used to detect the glucose content in the culture medium supernatant. In the presence of adenosine triphosphate (ATP) and magnesium ions, the glucose was phosphorylated by hexokinase (HK) to produce glucose-6-phosphate and adenosine diphosphate (ADP). The increase of absorbance at 340 nm was proportional to the glucose concentration in the sample.

### Glucose metabolism test

A CCK-8 kit was used to measure the cellular proliferation. HCCLM3 cells were plated in 96-well plates (8 × 10^3^/well) overnight, medium was changed to sugar-free medium the next day, after which the cells were left untreated or treated with Glucose (1 mM), Glucose (25 mM), 2-DG (1 mM), WZ35 (10 μg/mL). After an 18 h-drug treatment, the CCK-8 kit was used to measure the viability, with absorbance at 450 nm being quantified via microplate reader (Spectramax m5, Molecular Devices).

### Metabolomics analysis

TCA: Targeted metabolite analysis was performed on a Xevo G2-XS QT of mass spectrometry coupled with an ACQUITY UPLC (Ultra Performance Liquid Chromatography) system and data analysis was done with Progenesis QI (all from Waters, Milford, MA, USA). The metabolites in a homogenate of 1 × 10^6^ cells were extracted using chloroform, methanol, and water at a ratio of 8:4:3 and resuspended in 1% acetonitrile. For UPLC, the samples were injected on an HSS T3 column (100 × 2.1 mm, 1.8 μm) using an 8-min gradient containing flow phase A (0.1%formic acid–water) and flow phase B (0.1% formic acid-acetonitrile) at a flow rate of 0.5 ml/min. For the negative ion mode, the capillary energy and sample cone were set as 2000 V and 20 V, respectively. Scan time was set at 0.1 s intervals for 60 s.

Other metabolomics: A. 95% water: 5% acetonitrile + 10 mM ammonium acetate + NH_4_OH (PH = 10); B. 95% acetonitrile: 5% water + 10 mM ammonium acetate + NH_4_OH (PH = 10). Waters Acquity UPLC BEH Amide 2.1 × 100 mm, 1.7 μm. Time, flow velocity (mL/min), A1%, B1% were arranged in the following order: initial, 0.4, 1, 99; 0.1, 0.4, 1, 99; 6, 0.4, 70, 30; 6.5, 0.4, 1, 99; 10, 0.4, 1, 99.

### Tumor transplantation

Female BALB/c nude mice (6–8 weeks old; ~ 20 g) from Wenzhou Medical University Laboratory Animal Center were housed under SPF conditions. All procedures performed in studies involving humans and animals were in accordance with the ethical standards of the ethic committee of Wenzhou Medical University and with the 1964 Helsinki declaration and its later amendments or comparable ethical standards. Animals were subcutaneously injected with a 100 μL volume containing 5 × 10^6^ HCCLM3 cells in the right flank. When tumors grew to 100 mm^3^, the mice were randomized into 4 groups (n = 6/group) that were intraperitoneally administered saline, vehicle, curcumin (25 mg/kg), or WZ35 (25 mg/kg) daily for 17 consecutive days. Body weight and tumor volumes were monitored every other day, with the latter being calculated as follows: tumor volume = length × width × height/2. After 17 days, Intraperitoneal injection of 300 mg/kg sodium pentobarbital was used to euthanize mice. Upon examination, all mice lost heartbeat, breathing and righting reflex within two minutes and were therefore judged dead. The tumor tissues were collected to make paraffin sections for HE staining. Our project was conducted under guidelines approved by the Experimental Animal Ethics Committee of Wenzhou Medical University (ID number: wydw2019-0446).

In order to reflect the welfare and "3R" principles in animal experiments, and to "optimize" the animal experiment strategy, we set up "humane endpoints" to help animals reduce end-of-life pain and stress.Carry out detailed inspections at least twice a week, and increase the number of observations after the emergence of toxic manifestations:

General observations: rapid weight loss of 10–20%, loss of appetite or poor appetite, neurodepression with hypothermia without anesthesia, clinical symptoms of organ dysfunction and ineffective treatment, persistent effects of self-harm eating and drinking;Specific indicators: When the tumor growth volume exceeds 10% of the animal's body weight, the average diameter exceeds 15 mm, or the tumor surface ulcers or even causes infection or necrosis.In the event of any of the above symptoms, the experimenter will consider terminating the experiment immediately.

### Tissue microarrays (TMAs) and IHC staining

Liver cancer TMAs composed of 110 FFPE tissue Sects. (4 μm thick) were prepared the same way as previously described by Guttà C et al. [31] TMA IHC staining was conducted based on a standard approach. Briefly, the sections were deparaffinized using xylene, rehydrated with an ethanol gradient, treated to quench peroxidase activity, blocked with 5% normal goat serum, and incubated with anti-YAP/GLUT1 (1:200) overnight at 4 °C. As a negative control, the antibody was omitted. The slides were then probed with biotin-labeled goat anti-rabbit IgG, treated with a streptavidin peroxidase solution (SABC kit, Boster, Wuhan, China), and then 3,3-diaminobenzidine (Boster, Wuhan, China) in PBS with 0.05% H2O2 was used to treat the samples for 5 min at the room temperature for color development, after which the samples were counterstained using hematoxylin.

### IHC staining assessment

Two pathologists blinded to the samples evaluated the clinicopathological information independently and scored IHC images of TMA samples. We utilized the Image-Pro Plus 6.0 (IPP) (Media Cybernetics, MD, USA) to calculate the integrated optical density (IOD) for the stained images, with this value corresponding to total staining per sample area [32]. Entire TMAs were scanned in order to quantify protein levels in the TMA levels. IHC staining was evaluated based on immunoreactive score (IRS) values as described in the prior studies, with these scores reflecting both staining intensity and the percentage of the positive cells [25]. The percent positivity was stained as follows: 1 (≤ 10%), 2 (10–50%), 3 (50–80%), or 4 (≥ 80%). The staining intensity was scored as: 0 (negative), 1 (weak), 2 (moderate), or 3 (strong). These two scores were then multiplied together to yield the IRS value (0 – 12), with these values being deemed either low (≤ 6) or high (> 6).

### Statistical analysis

SPSS 19.0 (SPSS Inc., IL, USA) was utilized for statistical analysis. The data has been represented as the means ± SD from three independent experiments, and was compared via unpaired Student’s t-tests. The multi-group data were compared via Kruskal–Wallis test. Spearman's rank correlation coefficient test was applied to correlation analysis. Log-rank test was used for survival analysis. *P* < 0.05 was set as the significance threshold.

## Results

### YAP upregulation in liver cancer cells corresponds to poorer patient outcomes

**Fig. 1:**
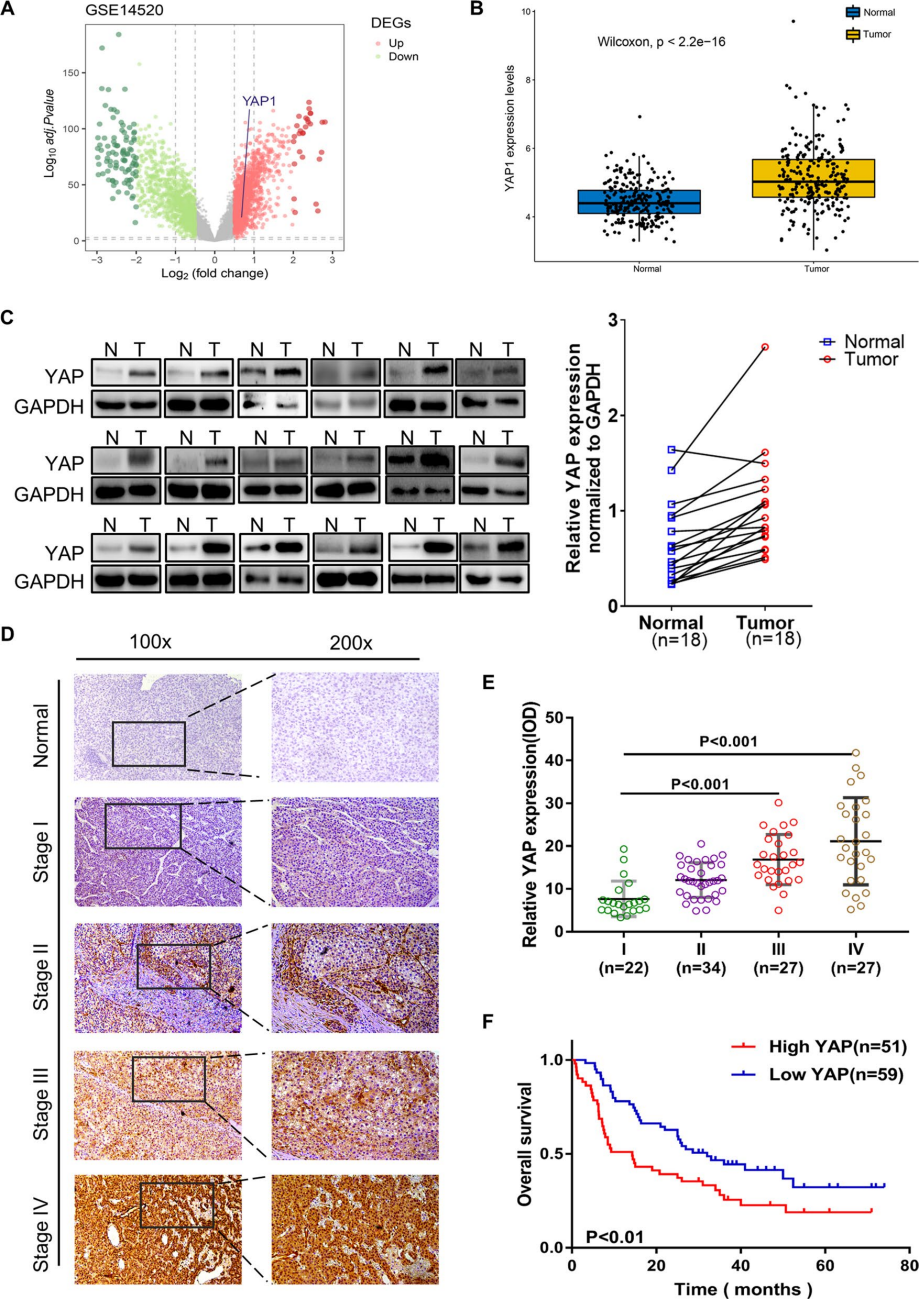
YAP upregulation in liver cancer cells corresponds to the poorer patient outcomes. **A** Volcano map of RNA-seq data from GEO database visualizing differentially expressed genes (DEGs) between the tumor samples and the normal ones with gene *YAP1* marked out (Fold change of > 0.5 and adjusted *P* < 0.05). **B** Box plot exhibits the distinct expression of *YAP1* in the normal and the tumor samples, with the blue box is representative of the tumor samples and the red box of the normal samples. **C** Western blotting shows differential expression of YAP in the normal and the tumor tissues. **D** and **E** YAP IHC staining in liver cancer patient samples. Representative YAP-stained images of normal, TNM stage I, II, III and IV tumor tissues have been shown and quantified. **F** Kaplan–Meier approach visualized the relationship between YAP expression and OS in liver cancer patients (n = 110, *P* < 0.01)

To understand the possible role of YAP in liver cancer, we began by analyzing data downloaded on Gene Expression Omnibus (GEO) datasets with accession number GSE14520. YAP1 is regarded the most common subtypes of YAP. Volcano map and Boxplots (Fig. 1A, B) reveal the upregulation of YAP1 in liver cancer cells (adjusted *P* < 0.001). The heat map visualizes the expression of all DEGs (Additional file 1: Fig.S1A). Visualization of enrichment analyses of up-regulated DEGs have been shown in Additional file 1: Fig. S1B.

The results of Western blotting on 18 liver cancer patients tissue samples and corresponding adjacent controls were assistant with the above (Fig. 1C). IHC staining composed of 110 patient-derived liver cancer samples shows that YAP expression has a positive association with tumor histological TNM stage (*P* < 0.001) (Fig. 1D, E). Moreover, the expression of YAP in well-differentiated (G1) liver cancer tissues was significantly lower than that were moderately (G2) (*P* = 0.003) and poorly ( G3) (*P* < 0.001) differentiated, and the levels of YAP were significantly higher in G3 samples in contrast to G2 samples (*P* < 0.001) (Additional file 1: Fig. S1C). These findings indicated that an upregulation of YAP can coincide with the progression of liver cancer. Kaplan–Meier analyses of 110 samples found that the high expression of YAP was closely related to poorer OS, while low related to better (Fig. 1F) (*P* < 0.01). In univariate analyses (Table1), the levels of YAP, tumor nodules, and venous invasion were all found to serve as significant prognostic factors, while in multivariate analyses, the expression of YAP was found to be independently associated with the prognosis of liver cancer patients. Together, these results indicated that YAP protein was overexpressed in liver cancer cells and may serve as an important target for therapeutic intervention.

**Table 1:** Univariate and multivariate analyses of factors associated with HCC patient overall survival

Variables	Univariate analysis				Multivariate analysis			
	RRb	SE	95%	*P* value	RRb	SE	95%	*P* value
YAP	1.819	0.233	1.151–2.875	0.01	1.717	0.235	1.083–2.721	0.021
Age	1.014	0.01	0.995–1.033	0.159	—	—	—	0.081
Gender	1.215	0.255	0.737–2.002	0.444	—	—	—	0.324
Tumor nodule	0.259	1.113	0.032–2.077	0.045	0.49	0.322	0.261–0.922	0.027
Lymph node metastases	0.899	0.232	0.570–1.416	0.645	—	—	—	0.762
HBV	0.985	0.149	0.736–1.318	0.917	—	—	—	0.724
Venous invasion	0.618	0.152	0.459–0.832	0.002	0.414	0.305	0.227–0.752	0.004
Liver cirrhosis	0.812	0.178	0.573–1.150	0.241	—	—	—	0.550

*YAP* yes-associated protein 1, *HCC* hepatocellular carcinoma, *HBV* hepatitis B virus, *SE* standard error

### WZ35 inhibited YAP from entering the nucleus and thereby affecting its function

**Fig. 2:**
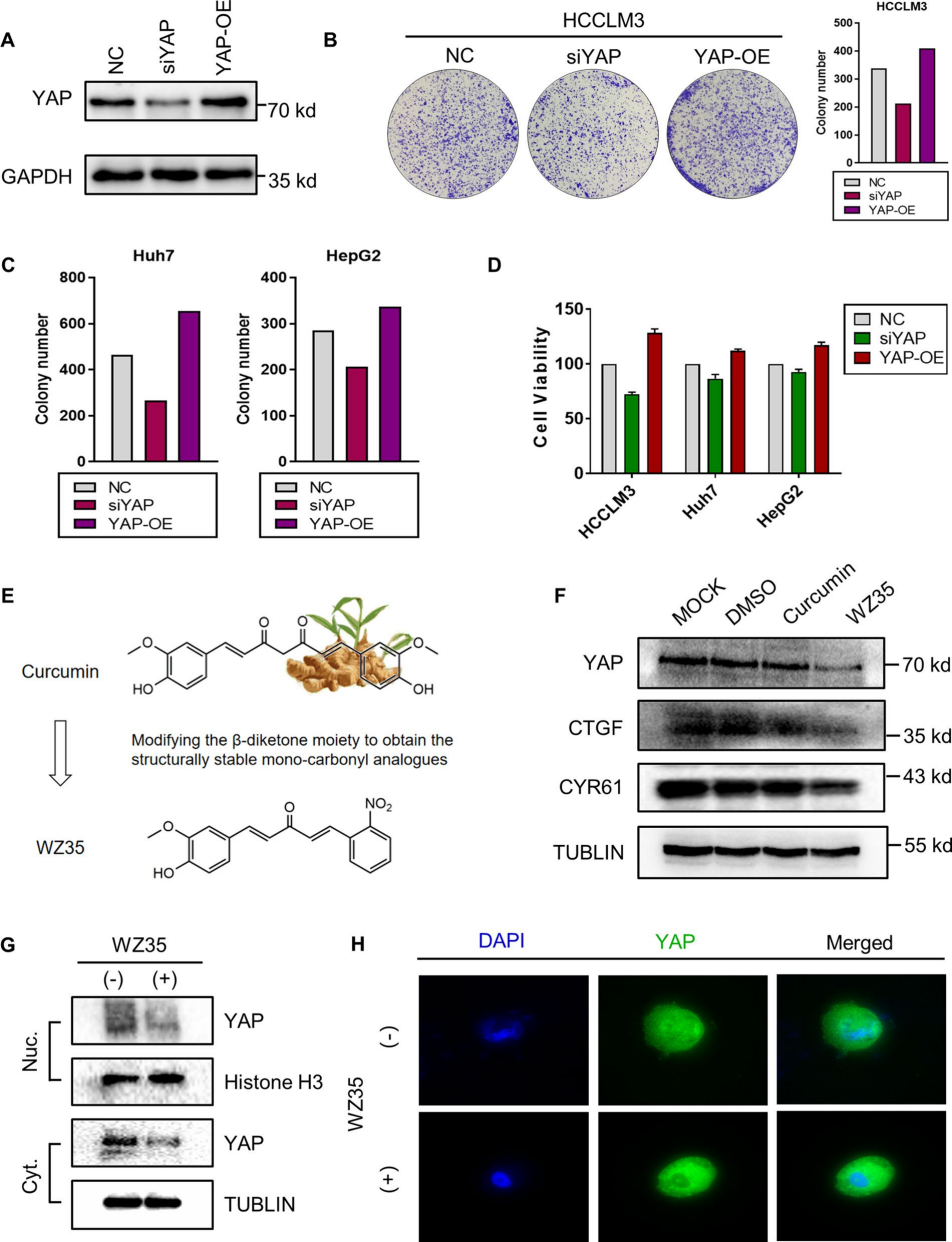
WZ35 can inhibit YAP from entering the nucleus. **A** HCCLM3 cell line was transfected with shRNA or pcDNA/peGFP vectors and harvested 48 h post-transfection to quantify the protein level of YAP and GAPDH. **B** and **C** Colony formation assay of different cell lines showed significant changes in stably transfected cells with representative images being shown and colony numbers being quantified. **D** CCK-8 assays were performed after cell lines were transfected with YAP shRNA or pcDNA/peGFP vectors. **E** The structure and synthesis of WZ35. **F** Western blotting analysis of the protein level of YAP, Cyr61 and CTGF in HCCLM3 cells treated with DMSO, curcumin and WZ35. **G** Western blot analysis of subcellular distribution of YAP in HCCLM3 cells treated with DMSO and WZ35.Histone H3 and Tublin are markers for nuclear and cytoplasm, respectively. Nuc., nucleus; Cyt., cytoplasm. **H** Representative immunofluorescent images via confocal microscopy were displayed with bars of 10 μm, confirming the reduction of nuclear localization of YAP protein. (Green: YAP; Blue: DAPI)

YAP protein was knocked down or over-expressed in HCCLM3 for various assays (Fig. 2A). YAP knockdown significantly reduced the colony formation rates, whereas the opposite facilitated this process (Fig. 2B, C). Moreover, consistent results were observed in a CCK-8 assay (Fig. 2D). These findings clearly suggested that the proliferation of liver cancer cells was regulated by YAP.

The structure of curcumin-derived WZ35 compound was shown in Fig. 2E. We proved that WZ35 can cause a significant downregulation of YAP and YAP-TEAD target genes Cyr61 and CTGF (Fig. 2F). Western blotting detected that WZ35 can decrease the total abundance and nuclear enrichment of YAP protein (Fig. 2G). Immunofluorescence assay observed that the green signals were significantly diminished following WZ35 treatment, particularly in the nuclei of liver cancer cells, with DAPI used for nuclear visualization (Fig. 2H), thereby indicating that WZ35 was able to retard the nuclear translocation of YAP. Overall, not only can WZ35 down-regulate YAP signaling, but can effectively hinder its translocation and ability to promote the viability of liver cancer cells.

### WZ35 showed superior anti-cancer activity under in vitro and in vivo settings

**Fig. 3:**
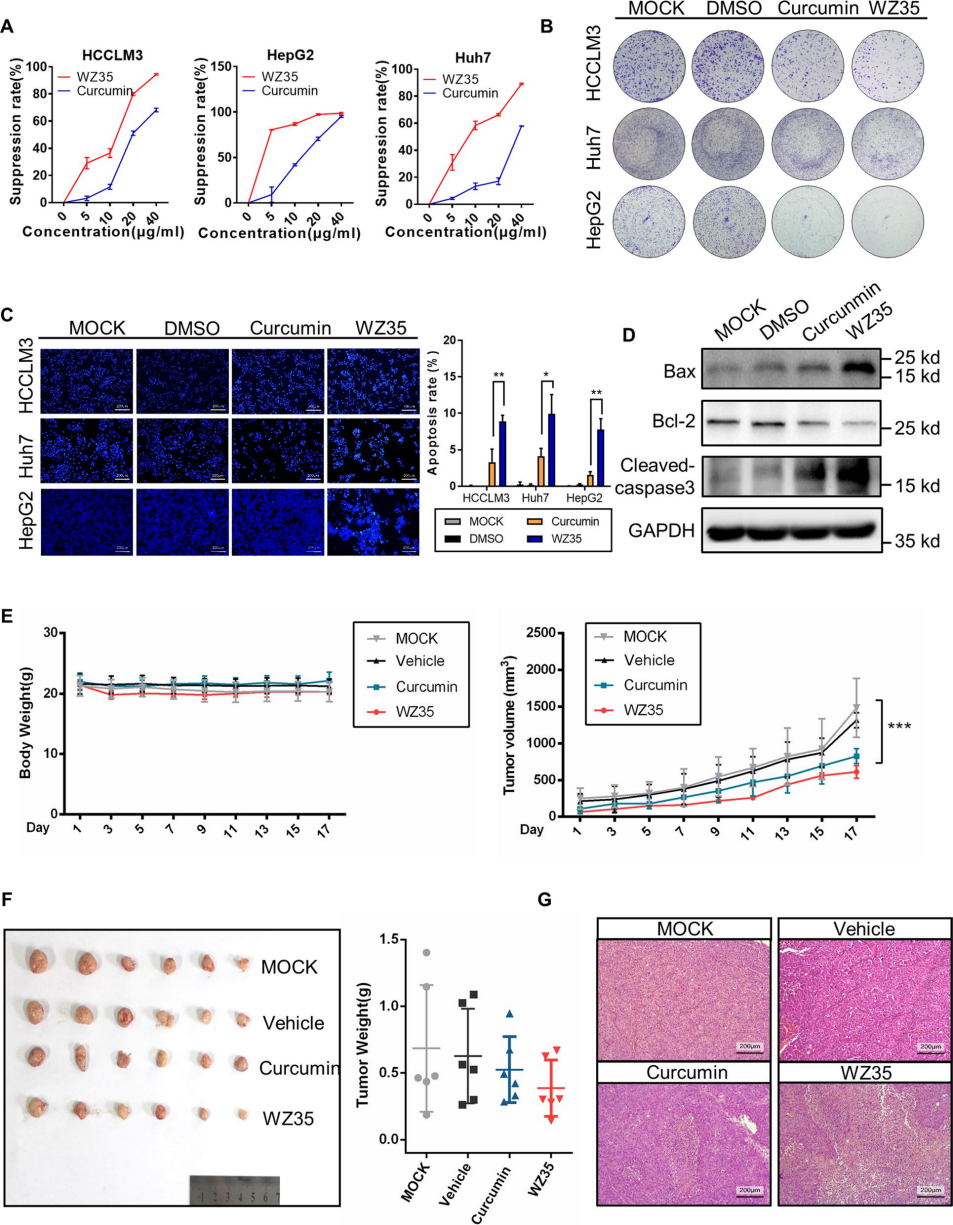
WZ35 exhibits significant antitumor activity. **A** CCK-8 assays were utilized to measure the suppression rate of curcumin and WZ35 (with the concentration of 0, 5, 10, 20 to 40 μg/mL) treated HCCLM3, HepG2, and Huh7 cells. **B** Colony formation assays of HCCLM3, HepG2, and Huh7 cells were conducted by plating 5000 indicated cells per well in 6-well plates to measure the effect of curcumin and WZ35 on clonogenicity with representative images and quantification of colony numbers being shown. **C** Representative fluorescence microscopy displaying nuclear transformations in HCCLM3, HepG2, and Huh7 cells following 18 h treatment of WZ35 (10 μg/mL) or treatment of curcumin (10 μg/mL) by means of DAPI staining. Results are presented as the mean ± standard error from independent experiments performed in triplicate. *P < 0.05, **P < 0.01, student’s *t* test. **D** The levels of various apoptosis associated proteins, including Bax, Bcl-2, and Cleaved caspase-3 were detected by western blotting following the 18 h treatment of WZ35, with GAPDH serving as an internal control. **E** Quantitation of the murine body weight and tumor volumes over time in four indicated tumor xenograft groups, as measured by utilizing electronic balance and caliper. The results are presented as the mean ± standard error from independent experiments in triplicate. *P < 0.05, **P < 0.01, ***P < 0.001, student’s *t* test. **F** Subcutaneous tumor xenograft models of WZ35-treated HCCLM3 were used. Mice were put to death by cervical dislocation and the tumors were harvested after 17 days. Optical image of each tumor and the weight curve was photographed and quantified, respectively. (n = 6 mice per group). **G**) The representative images of Hematoxylin and eosin staining of indicated treated tumors have been shown. Scale bars, 200 μm for images

In a CCK-8 assay, both curcumin and WZ35 caused a dose-dependent inhibition of the tumor cell proliferation, with WZ35 being superior to curcumin (Fig. 3A). Moreover, similar outcomes were observed in colony formation assays (Fig. 3B, Additional file 2: Fig. S2A). Flow cytometry analysis indicated that WZ35 substantially induced G2/M cell cycle arrest (Additional file 2: Fig. S2B), thus suppress the growth of HCCLM3 cells, and promoted apoptosis (Additional file 2: Fig. S2C). DAPI staining revealed that WZ35 treatment induced nuclear shriveling and abnormal nuclear morphology at higher rates as compared to curcumin (Fig. 3C). Western blotting detected that drug-treated cells exhibited an upregulated levels of Bax and cleaved caspase-3 and a decrease of Bcl-2 (Fig. 3D).

In order to extend our range of findings to in vivo settings, nude mice bearing subcutaneous HCCLM3 tumor xenografts were used. Both curcumin and WZ35 treatment did not significantly alter murine body weight (Fig. 3E). While both the compounds were able to inhibit tumor growth, WZ35 was found to be superior in suppressing this growth over a 17-day study period (Fig. 3E, F). In histological assessment, WZ35-treated tumors revealed lower cellularity and higher tumor necrosis rates as compared with the vehicle and curcumin treatment (Fig. 3G). These results suggest that WZ35 may exhibit preferable in vivo efficacy by markedly suppressing tumor growth as compared to curcumin.

### WZ35 inhibits liver cancer growth in a ROS-dependent manner

**Fig. 4:**
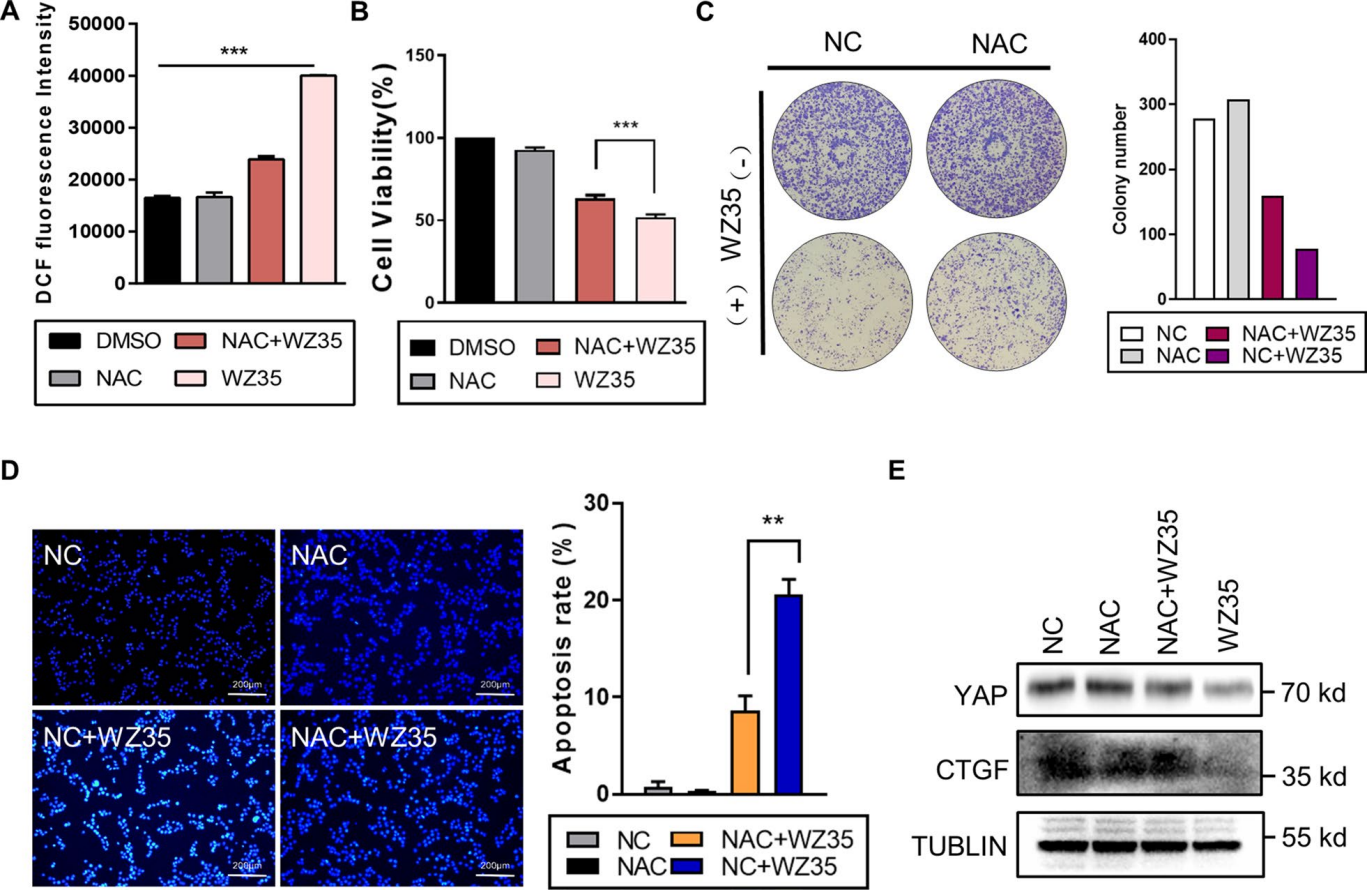
WZ35 inhibits growth of liver cancer cells in a ROS-dependent manner. **A** The levels of intracellular reactive oxygen species (ROS) were determined by measuring the mean fluorescence intensity (MFI) of DCFH-DA (DCF) via flow cytometry in control or WZ35-treated HCCLM3 cells following pretreatment with NAC. Results are presented as the mean ± standard error from independent experiments in triplicate. ***P < 0.001, student’s *t* test. **B**–**D** HCCLM3 cells with or without NAC (5 mM) pretreatment were incubated with or without WZ35 (10 μg/mL) to assess the cellular proliferation via CCK-8 assays **B** and colony formation assays **C**, assess cellular apoptosis via DAPI staining assays **D** with representative images and/or quantifications have been shown. **E** Western blotting analysis of the protein level of YAP and CTGF in HCCLM3 cells treated with NAC and WZ35. All these results are presented as the mean ± standard error from independent experiments in triplicate. **P < 0.01, ***P < 0.001, student’s *t* test

To further ascertain the cellular mechanism related to drug-associated apoptosis, we examined the impact of WZ35 on intracellular morphology in HCCLM3 cells via TEM. Apparent mitochondrial dysregulation, scattering, and swelling were observed in the treated cells (Additional file 2: Fig. S2D). Mitochondria are the major intracellular sources of ROS generation owing to electron leakage from the electron transport chain (ETC). We therefore analyzed ROS levels via flow cytometry. This treatment led to a more than twofold increase in intracellular ROS levels (Fig. 4A). It’s widely known that excessive ROS levels can cause deleterious oxidative damage [33]. Hence, an increased ROS generation observed in WZ35-treated cells was likely to contribute to observed reduction in the cellular proliferation.

Thereafter, we utilized N-acetyl-l-cysteine (NAC) to inhibit ROS before WZ35 treatment. NAC pretreatment neutralized WZ35-induced cells proliferation inhibition (Fig. 4B), and correspondingly facilitated the colony forming abilities of WZ35-treated cells (Fig. 4C) while attenuating their apoptotic death (Fig. 4D). Western blotting revealed that this antioxidant can significantly counteract changes in the expression of YAP and its target gene CTGF caused by WZ35 (Fig. 4E).

All these results suggested that ROS generation was essential in order for WZ35 to suppress growth and proliferation, with the expression of YAP being closely linked to increased ROS production in treated liver cancer cells.

### WZ35 antitumor activity depends upon inhibition of YAP and allows it to regulate GLUT1 expression

**Fig. 5:**
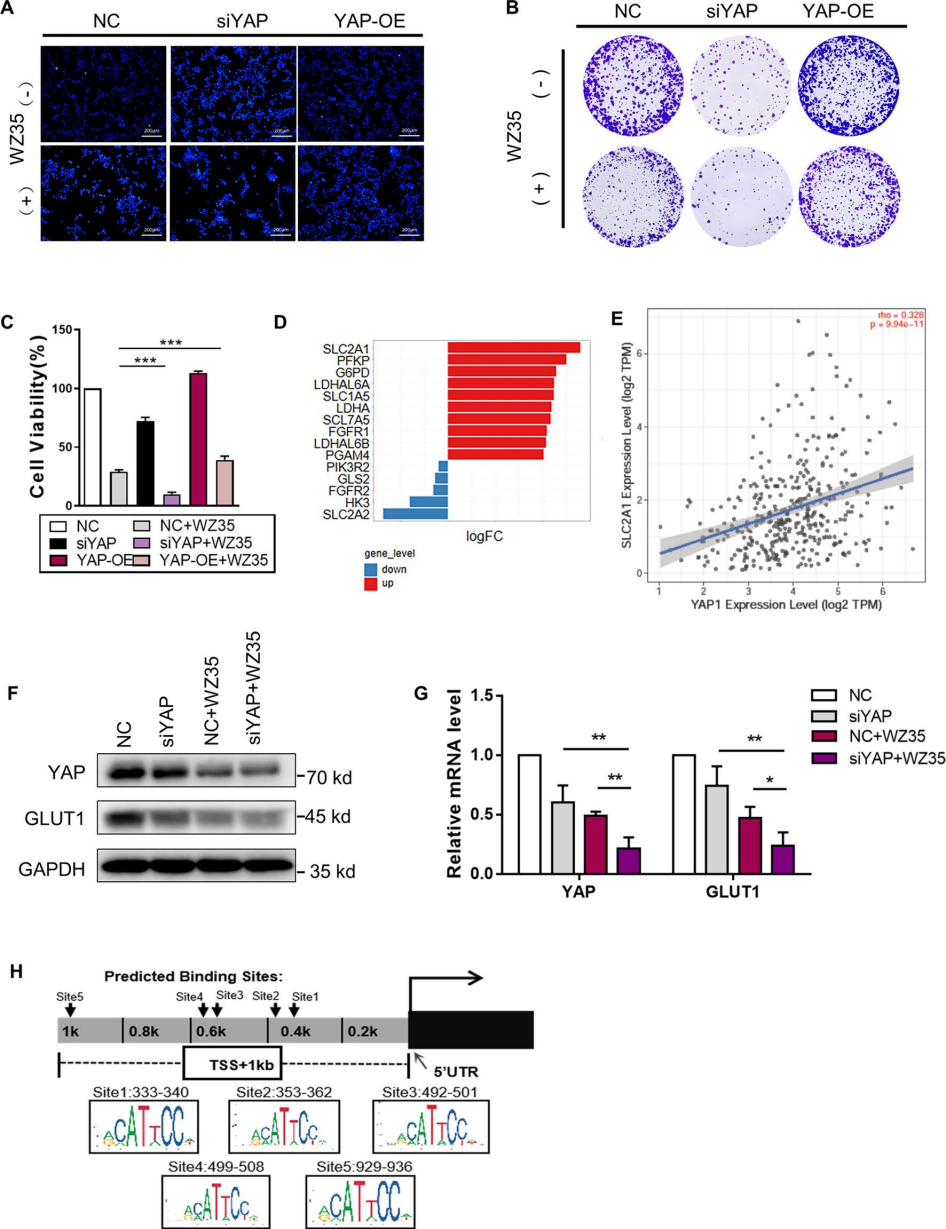
WZ35 antitumor activity depends upon inhibition of the YAP protein and allows it to regulate the expression of GLUT1. **A**–**C** DAPI staining assays **A**, colony formation assays **B** and CCK-8 assays **C** were conducted to measure apoptosis and cellular viability in control or WZ35-treated (10 μg/mL) HCCLM3 cells with or without the pretreatment of knockdown or overexpression via shRNA or pcDNA/peGFP vectors. **D** Barchart visualized significantly related genes in central carbon metabolism with higher YAP1 expression including 10 genes with the highest logFC and 4 genes with the lowest logFC from datasets GSE97098, GSE77314 and GSE153783. **E** Correlation between *YAP1* and *SLC2A1* in liver cancer samples from TIMER dataset (*P* = 9.94 × 10^–11^) was visualized in a scatter diagram. **F** Western blotting analysis of the YAP and GLUT1 protein level of HCCLM3 cells treated with WZ35 and plasmid. **G** Real-time qPCR analysis of the YAP and GLUT1 mRNA level of HCCLM3 cells treated with WZ35 and plasmid. **H** Predicted TEAD binding motif site sequence in the promoter region of *GLUT1* host gene chromosome 1 from the database JASPAR

YAP was knocked down and over-expressed in HCCLM3 cells. The results of various assays indicated that WZ35 and downregulation of YAP caused a concurrent inhibitory effect on cell viability (Fig. 5C) and clonogenicity (Fig. 5B, Additional file 3: Fig. S3B), therefore facilitating death (Fig. 5A, Additional file 2: Fig. S3A).

Data downloaded from TCGA was divided into two groups according to *YAP1* expression. Enrichment analysis based on Gene Ontology (GO) annotation system was conducted. The samples were divided into YAP1 high-expression and YAP1 low-expression groups according to the median of YAP1 expression. The DEGs correlated with YAP1 expression had enriched GO terms related to metabolism (Additional file 3: Fig. S3C). Correlated DEGs were also mainly enriched in oncogenic pathway for liver cancer cells (Additional file 4: Fig. S4). As shown in Fig. 5D, E, SLC2A1 expression of YAP1 high expression group was significantly up-regulated and exhibited high association with YAP1. Meanwhile, the pathview (Additional file 5: Fig. S5) showed the changes of genes expression related to central carbon metabolism in the YAP high-expression group versus the low-expression group. SLC2A1 is the official name of GLUT1. Bioinformatics analysis of GEO database proved that YAP expression may be closely related to cellular metabolism genes especially the genes in glycolysis process (Additional file 6: Fig. S6A and B). GLUT1 was significantly up-regulated in tumor samples (Additional file 7: Fig. S7A and B) and high levels of it was associated with a shorter survival in liver cancer patients (Additional file 7: Fig. S7C). Western blotting and qPCR analysis unfolded that WZ35 decreased GLUT1 expression and that knockdown of YAP enhanced the suppressive effect of WZ35 on GLUT1 (Fig. 5F-G), whereas overexpression of YAP neutralized it (Additional file 7: Fig. S7D). Based on the JASPAR [25] database, five potential TEAD binding sites were present on the GLUT1 promoter (Fig. 5H). UCSC Genome Browser revealed that GLUT1 promoter is highly conserved among mammals and an increased level of histone methylation correlated with H3K4Me3 at the promoter (Additional file 8: Fig. S8).

### WZ35 might mediate the inhibition of glucose intake to reduce proliferation

**Fig. 6:**
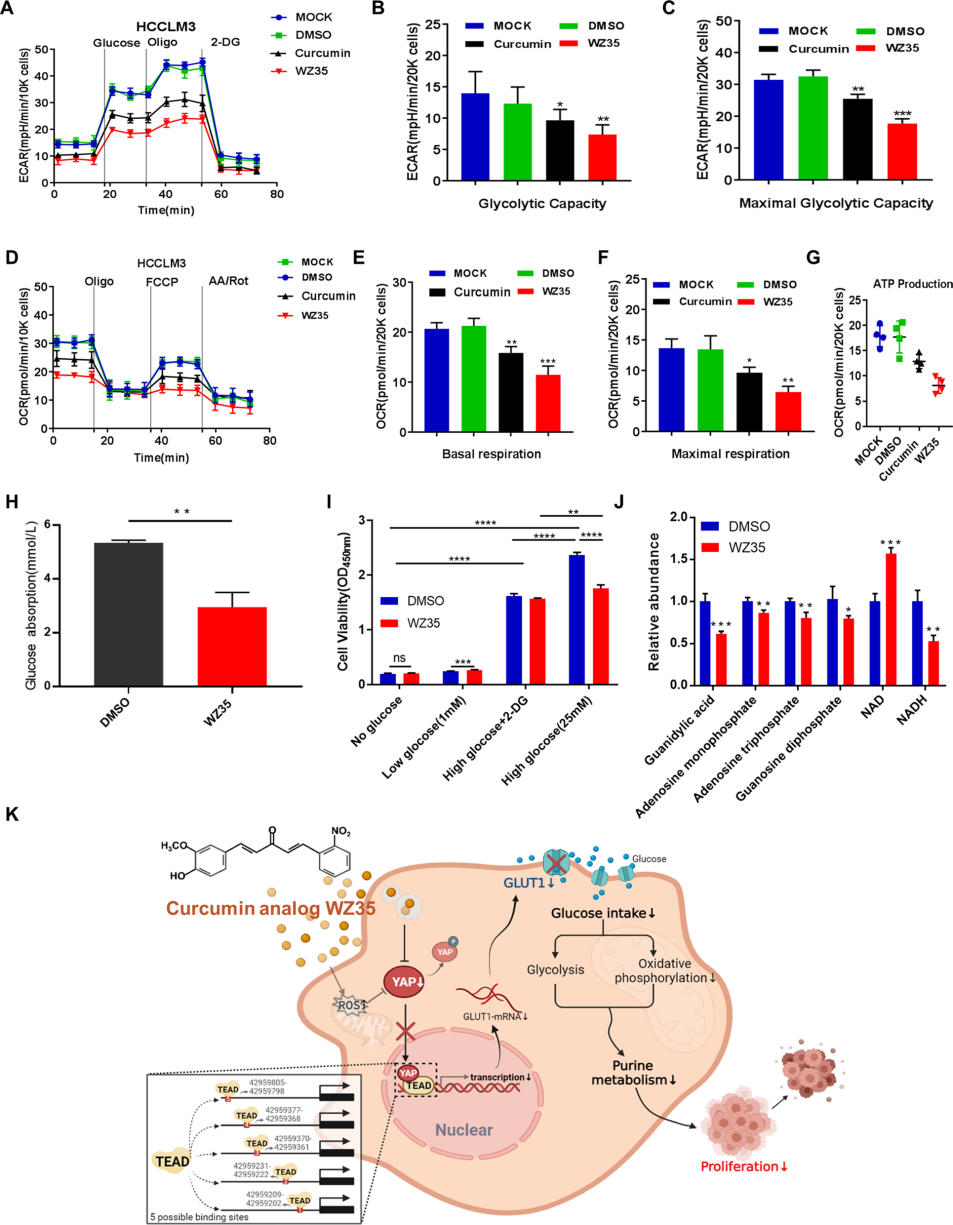
WZ35 caused a significant disturbance in the cellular metabolic state. **A** and **D** ECAR and OCR was measured with Seahorse XF96 Flux analyzer in HCCLM3 cells. **B** and **C** Glycolytic capacity and maximal glycolytic capacity reserve were measured in non-glucose medium after the addition of glucose (10 mM), oligomycin (1 μM) and 2-DG (100 mM). **E**–**G** Basal respiration, maximal respiration and ATP production were measured after the injection of oligomycin (1 μM), FCCP (0.5 μM) and AA/Rot (2 μM). **H** The glucose concentration of culture medium was detected by hexokinase method. **I** The absorption of glucose was measured in combination with CCK-8 assay in either non-glucose-treated or low glucose-treated or high glucose-treated or high glucose plus 2-DG-treated HCCLM3 cells with or without treatment of WZ35. **J** The contents of metabolic products in HCCLM3 cells treated with or without WZ35 (10 μg/mL). All these results have been presented as the mean ± standard error from independent experiments in triplicate. *P < 0.05, **P < 0.01, ***P < 0.001, ****P < 0.0001, student’s *t* test. **K** Proposed working model showed distinct metabolic changes after drug action

Statistically, after action of curcumin or WZ35, both two curves of the total acidification rate moved down, and the descending degree of the latter outweighed that of the former (Fig. 6A–C), so did the curves of OCR (Fig. 6D–G). We then detected the absorption of glucose via glucose determination kit (hexokinase method). WZ35 was found to significantly attenuate the ability to absorb glucose from outside obviously (Fig. 6H). By means of CCK-8 assay (Fig. 6I), the absorption of glucose was correlated to the proliferative activity of cells. When we artificially retarded the glycolysis process, the ability of WZ35 to inhibit cell proliferation was reduced, which could possibly exert its anti-proliferative effects by hindering the process of cell glycolysis. Metabolomics was used to detect the levels of the various intracellular metabolites. As displayed in Fig. 6J, WZ35 enhanced the ratio of NAD^+^ to NADH, signifying the blockade of oxidative phosphorylation. The content of purine metabolites were also significantly attenuated. The general metabolism changes triggered by WZ35 have been shown in Fig. 6K.

### YAP combined GLUT1 may serve as a valuable prognostic and/or diagnostic biomarker in liver cancer

**Fig. 7:**
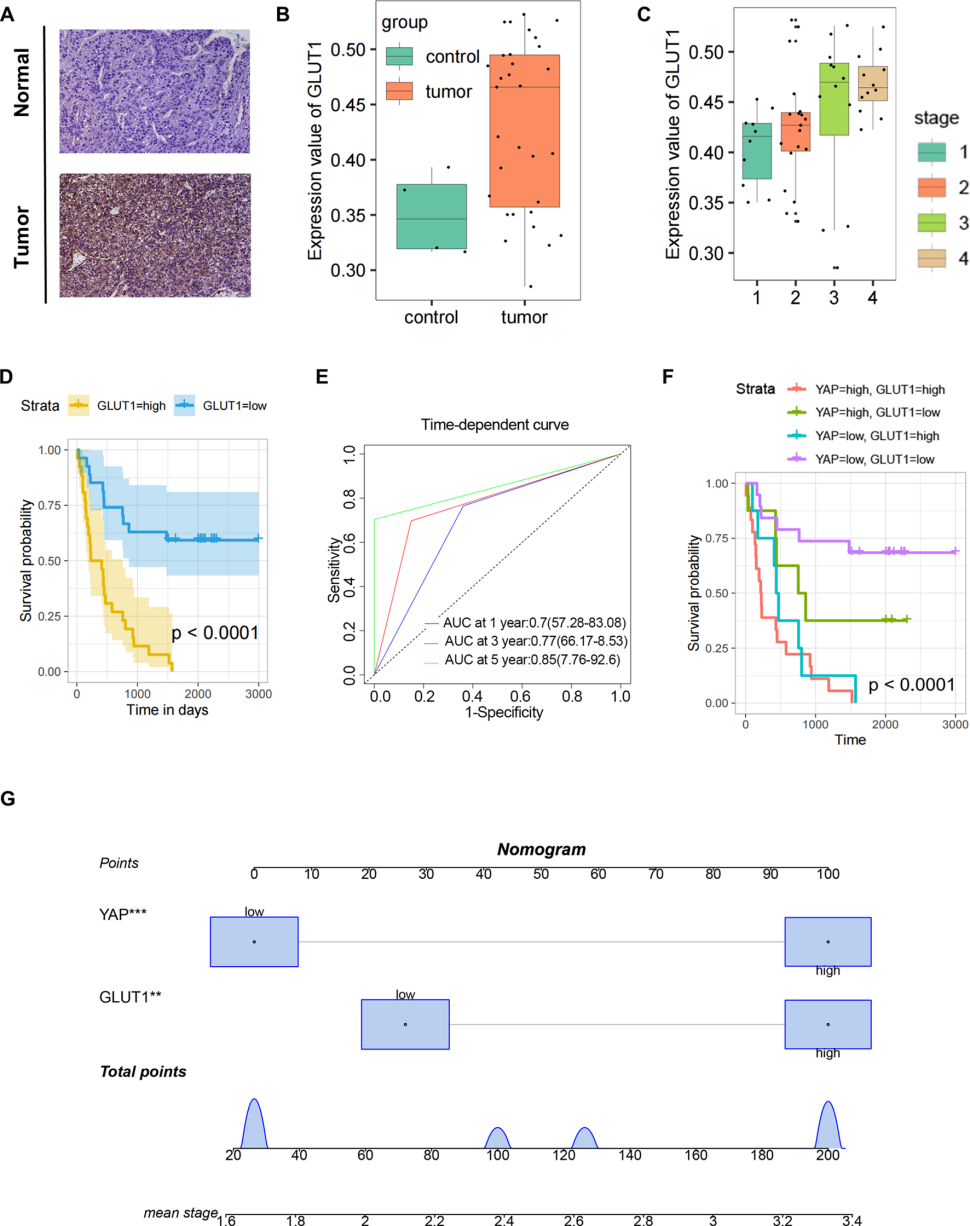
YAP combined GLUT1 may serve as a valuable prognostic and/or diagnostic biomarker in liver cancer. **A** and **B** GLUT1 IHC staining in liver cancer patient samples. Images of cells were used to evaluate GLUT1 expression between the normal and the tumor. **C** GLUT1 IHC staining in liver cancer patient samples. Representative GLUT1-stained images of TNM stage I, II, III and IV liver cancer tumor tissues were quantified. **D** Kaplan–Meier approach visualized the relationship between GLUT1 expression and OS in liver cancer patients (n = 53, *P* < 0.001) **E** Time-dependent ROC curves analyzing the potential value of expression levels of GLUT1. **F** Kaplan–Meier curve for the disease free survival and overall survival have been illustrated for the subgroups YAP high/GLUT1 low, YAP high/GLUT1 high, YAP low/GLUT1 low and YAP low/GLUT1 high. **G** Nomogram for predicting the stage according to the expression levels of GLUT1 and YAP. Instruction: Each characteristic was located on the corresponding variable axis, and a vertical line was drawn upwards to the points axis to determine the specific point value. This process was repeated and the total points value was tallied and located on the linear pretector axis. A vertical line was drawn down to the rank to obtain the probability of later stage

We further explored clinical application of YAP and GLUT1 in above tissue chip with 53 liver cancer samples. The majority of these liver cancer patients were GLUT1-positive (Fig. 7A, B) (*P* = 0.0208). Boxplot showed that GLUT1 expression was positively correlated with TNM stage (Fig. 7C), suggesting that GLUT1 upregulation could coincide with liver cancer progression. Kaplan–Meier survival analysis showed that high level of GLUT1 was associated with a shorter survival (Fig. 7D) and the areas under curve (AUC) in ROC curve of GLUT1 were 0.7 (at 1 year), 0.77 (at 3 years) and 0.85 (at 5 years) (Fig. 7E). Since biological information mining found that YAP and GLUT1 might display a good predictive function in predicting the prognosis of liver cancer patients (Additional file 9: Fig. S9A-D), to further substantiate the survival significance of YAP and GLUT1, the correlation between YAP/GLUT1 high expression and clinical features of liver cancer patients was analyzed in above 53 clinical cases. The expression of YAP was found to be proportional to GLUT1 (Additional file 9: Fig. S9E). High integrated expression levels of YAP and GLUT1 were negatively correlated with survival in liver cancer patients (Fig. 7F). A nomogram was made according to the result of multi-variate logistic proportional hazards analysis which considers Stage I and II as the early stage, Stage III and IV as the late stage. Through fixing the points associated with YAP and GLUT1, the risk of later stage for an individual patient could be calculated (Fig. 7G). A ROC calibration plot was made to assess the internal validity for the constructed nomogram (Additional file 9: Fig. S9F).The AUC for combination of YAP and GLUT1 was 0.889, which was higher than GLUT1 alone (Additional file 9: Fig. S9G). Considering these data, we can infer the potential link between YAP and GLUT1 and the integration between them may potentially serve as a valuable prognostic biomarker in liver cancer patients.

## Discussion

Liver cancer is the second deadliest and fifth most prevalent cancer type globally in 2020 [1]. However, the onset of liver cancer is insidious, and once discovered, it is mostly diagnosed an advanced stage. Therefore, identification of efficient treatment and treatment targets for liver cancer has become an urgent problem [34].

WZ35, developed by our team, is an analog of curcumin that can suppress the growth of variety of tumors by targeting YAP [23–26]. One of our previous study [24] found that WZ35 could inhibit glycolysis. We further discovered that WZ35 blocked glucose absorption and investigated the metabolic changes in hepatocellular carcinoma cells after that through metabolomics. According to the desperate glucose demanding of cancer cells, some scholars suggested that intermittent fasting might diminish the progression of tumors [35]. Clinical data showed that fasting can prevent DNA injury and immune cells damage induced by chemotherapy [36]. Combining fasting with other therapies can prevent drug resistance and reduce side effects [37]. However, the lack of nutrition caused by fasting makes the overall anti-cancer effect unsatisfactory. For example, the reduction of glutamine due to glucose deficiency can alleviate immune infiltration in the tumor microenvironment [38]. Fasting might induce more diseases such as anemia, neurocognitive deficits [39], etc. In this study, we demonstrated that the glucose blocking ability of WZ35 is one of its main anti-cancer mechanisms (Fig. 6I). And that suggests, WZ35 has the same anti-cancer effect as intermittent fasting, and it also cleverly avoids serious side effects caused by the deficiency of other nutrition, which makes WZ35 a promising candidate drug for cancer therapy.

To further elucidate the underlying mechanism, we investigated the WZ35-induced inhibition of glucose uptake at the molecular level. Our study revealed that WZ35 can disrupt ROS homeostasis and downregulate the YAP signal in nucleus through reducing the total protein content and blocking its translocation from cytoplasm to nucleus. Although the role of YAP in organ regeneration [40, 41] and atherosclerosis [42] has been well-studied, how YAP takes part in glucose mechanism remains unknown. In this study, we demonstrated that WZ35 affected the expression of GLUT1 to impair glycolysis via regulating YAP expression in hepatocellular carcinoma cells. GLUT1, as the primary glucose transporter, is considered to be the most highly conserved and widely found glucose transporter in different cancers [8]. A recent study showed that endogenous TEAD plays an essential role in mediating GLUT1 expression and upregulating glycolysis in the progress of cardiac hypertrophy [43]. Based on our results above, we reasonably believed that the YAP-TEAD complex formed on the distal promoter or the enhancer of the GLUT1 gene may involve in the interaction mechanism between YAP and GLUT1. H3K4me3 is positively associated with gene expression and is regarded as co-factors of translation modifications which always gathers around transcription start sites [44]. By analyzing JASPAR database, we predicted five potential TEAD-binding sites in the promoter region of GLUT1. Four of them were found to have increased level of histone modifications associated with H3K4me3. It Increased credibility of the hypothesis that in hepatocellular carcinoma cells, YAP may mediate glycolysis through YAP-TEAD-GLUT1 pathway. Nevertheless, this hypothesis requires more direct and convincing evidences in future investigations.

To further explore potential clinical applications of the experimental data, we assessed the relationship of YAP and GLUT1 expression levels in hepatocellular carcinoma tissue with clinicopathologic features of HCC patients. Multi-variate Cox proportional hazards analysis testified that YAP and GLUT1 has important reference significance for predicting the stages of disease progression in liver cancer patients. It suggested that YAP and GLUT1 may be promising biomarkers in HCC for diagnosis, treatment and prognosis.

We next explored the role glucose uptake inhibition taken in cellular metabolic network. Increased glucose uptake is seen as the main characteristic of tumor cells. Glycolysis and oxidative phosphorylation take on critical roles in glucose metabolism [45].

This study revealed that WZ35-induced relative glucose shortage restrained glycolysis and oxidative phosphorylation. And our metabolomics analysis showed that WZ35 can inhibit purine metabolism. Many intermediates in glycolysis can be precursors of different biosynthetic procession. For example, 5-phosphate ribose, the structural component of nucleotides, is partially oxidized from 6-phosphate glucose (PPP) [46]. Current studies suggested that the level of oxidative phosphorylation is closely related to proliferation ability [47, 48]. Generation of the nucleotide bases in purine nucleotide metabolism can promote the proliferation of glutamine-deficient cells [49, 50]. Thus, we speculated that WZ35 may block glycose uptake to inhibit the progress of both glycolysis and oxidative phosphorylation, thereby decreases the proliferation activity of HCCLM3.

In conclusion, we demonstrated that, compared with curcumin, WZ35 reveals superior antitumor activity. It mediates ROS/YAP/GLUT1 loop to inhibit glucose uptake and oxidative phosphorylation, which ultimately decreases the proliferation activity of liver cancer cells. These findings may deepen our understanding regarding the ROS/YAP/GLUT1 loop in HCC metabolic process. Besides, YAP-GLUT1 has reference significance in predicting the stages of progression in HCC. It is promising to become novel biomarkers for the diagnosis and treatment of liver cancer.

## Supplementary Information

**Additional file 1: Figure S1. **(A) Differential gene expression between liver cancer and the control tissues in the GSE14520 dataset was analyzed, with the patterns of differential gene expression (adjusted *P* < 0.05, ∣logFC∣≥ 0.5) being organized into a heat map. (B) Gene Ontology (GO) and Kyoto Encyclopedia of Genes and Genomes (KEGG) enrichment analysis of DEGs of tumor samples with normal ones. (C) YAP IHC staining in liver cancer patient samples. Representative YAP-stained images of Grade 1 (G1), Grade 2 (G2), and Grade 3 (G3) liver cancer tumor tissues have been quantified.

**Additional file 2: Figure S2.** (A) Quantifications of Colony formation assays. (B) Flow cytometry analysis was utilized in the control or WZ35-treated HCCLM3 cells to measure the possible arrest in the cell cycle. Representative fitting curve of the cell cycle qualitatively illustrated the percentage of cells in G1, S and G2/M phase. (C) Apoptosis was assessed via flow cytometry in HCCLM3 cells with or without the treatment of WZ35 (10 μg/mL) for 18h using Annexin V-FITC/PI double staining, visualized by scatter plots. Percentages of apoptosis were quantified and the results have been presented as the mean ± standard error from independent experiments in triplicate. ***P < 0.001, student’s* t* test. (D) Transmission electron micrographic (TEM) imaging showed changes in the morphology of HCCLM3 cells subsequent to the treatment of curcumin (10 μg/mL) or WZ35 (10 μg/mL) respectively.

**Additional file 3: Figure S3. **(A and B) Representative images and/or quantifications of DAPI staining assays (A) and colony formation assays (B) have been shown. All these results are presented as the mean ± standard error from independent experiments in triplicate. **P < 0.01, ***P < 0.001, student’s *t* test. (C ) Enriched gene ontology (GO) (C) involved by YAP expression related genes.

**Additional file 4: Figure S4. **(A) KEGG pathways involved by YAP expression related genes.

**Additional file 5: Figure S5. **Genes in central carbon metabolism pathway of YAP high expression group on KEGG graph rendered by pathview. Gene expression levels are indicated as significantly higher (red), unchanged (gray), or lower (green).

**Additional file 6: Figure S6. **(A) Heatmap visualizing DEGs' in liver cancer samples from TCGA datasets. (B) Heatmap visualizing glucose metabolism genes' expression levels exposed to different YAP1 expression in liver cancer samples from TCGA datasets.

**Additional file 7: Figure S7.** (A) Volcano plot of RNA-seq data from GEO database with *SLC2A1 *being marked out. (B) Box plot exhibits the distinct expression of *SLC2A1* in the normal and the tumor samples, with the blue box being the representative of the tumor samples and the red box of the normal samples. (C) Kaplan–Meier analysis displaying survival for liver cancer patients stratified by expression levels of *SLC2A1* (*P *= 3.7×10^-6^). (D) Western blotting analysis of the YAP and GLUT1 protein level of HCCLM3 cells treated with WZ35 and plasmid.

**Additional file 8: Figure S8. **The evolutionary conservation of GLUT1 promoter and the modified state of histone methylation were depicted using the University of California, Santa Cruz (UCSC) genome browser.

**Additional file 9: Figure S9.** (A and C) Time-dependent ROC curves analyzing the potential value of expression levels of *SLC2A1* (A) and combination of *SLC2A1* and *YAP1* (C) in diagnosis of liver cancer. (B and D) Kaplan–Meier analysis displaying survival rate for liver cancer samples from GEO datasets according to the expression levels of single factor of *SLC2A1* (B) (*P* = 0.00035) and multi factors of *YAP1* and *SLC2A1* (D) (*P* < 0.0001). High integrated expression levels of *YAP1* and *SLC2A1* were related to the poor survival and prognosis. (E) Kaplan-Meier approach visualized the relationship between the expression of YAP and OS in liver cancer patients (n = 53, *P* = 0.0018). (F) ROC calibration plots of the nomogram for combined YAP and GLUT1：The AUC was 0.889. (G) ROC calibration plots of the nomogram for GLUT1: The AUC was 0.775.

**Additional file 10: Table S1 **The table listing about the 1348 upregulated genes in HCC patients.** Table S2.** The table listing the upregulated genes based on YAP-related subgroup analysis of HCC patients.

## Data Availability

The datasets used and/or analyzed during the present study are available from the corresponding author on reasonable request.

## Abbreviations

YAP: Yes-associated protein 1
YAP1: Yes1 associated transcriptional regulator
GLUT1: Glucose transporter 1
TEM: Transmission electron microscope
SLC2A1: Solute carrier family 2
ROS: Reactive oxygen species
OCR: Oxygen consumption rate
ECAR: Extracellular acidification rate
TCGA: The Cancer Genome Atlas
GEO: Gene Expression Omnibus
TCA cycle: Tricarboxylic acid cycle
NAD +: Oxidized nicotinamide adenine dinucleotide
ROC: Receiver operating characteristic
AUC: Area under curve
OS: Overall survival
IHC: Immunohistochemistry
TNM: Tumor node metastasis
DAPI: 4′,6-Diamidino-2-phenylindole
GFP: Green fluorescent protein
FBS: Fetal bovine serum
PBS: Phosphate-buffered saline
SDS-PAGE: Sodium dodecyl sulfate polyacrylamide gel electrophoresis
SPF: Specific-pathogen-free
H&E: Hematoxylin and eosin
DEG: Different expression gene
