# Supplementary figures and images for: A novel small molecule glycolysis inhibitor WZ35 exerts anti-cancer effect via metabolic reprogramming
Source: J Transl Med. 2022 Nov 18;20:530. doi: 10.1186/s12967-022-03758-0 (PMC9673307; doi:10.1186/s12967-022-03758-0)

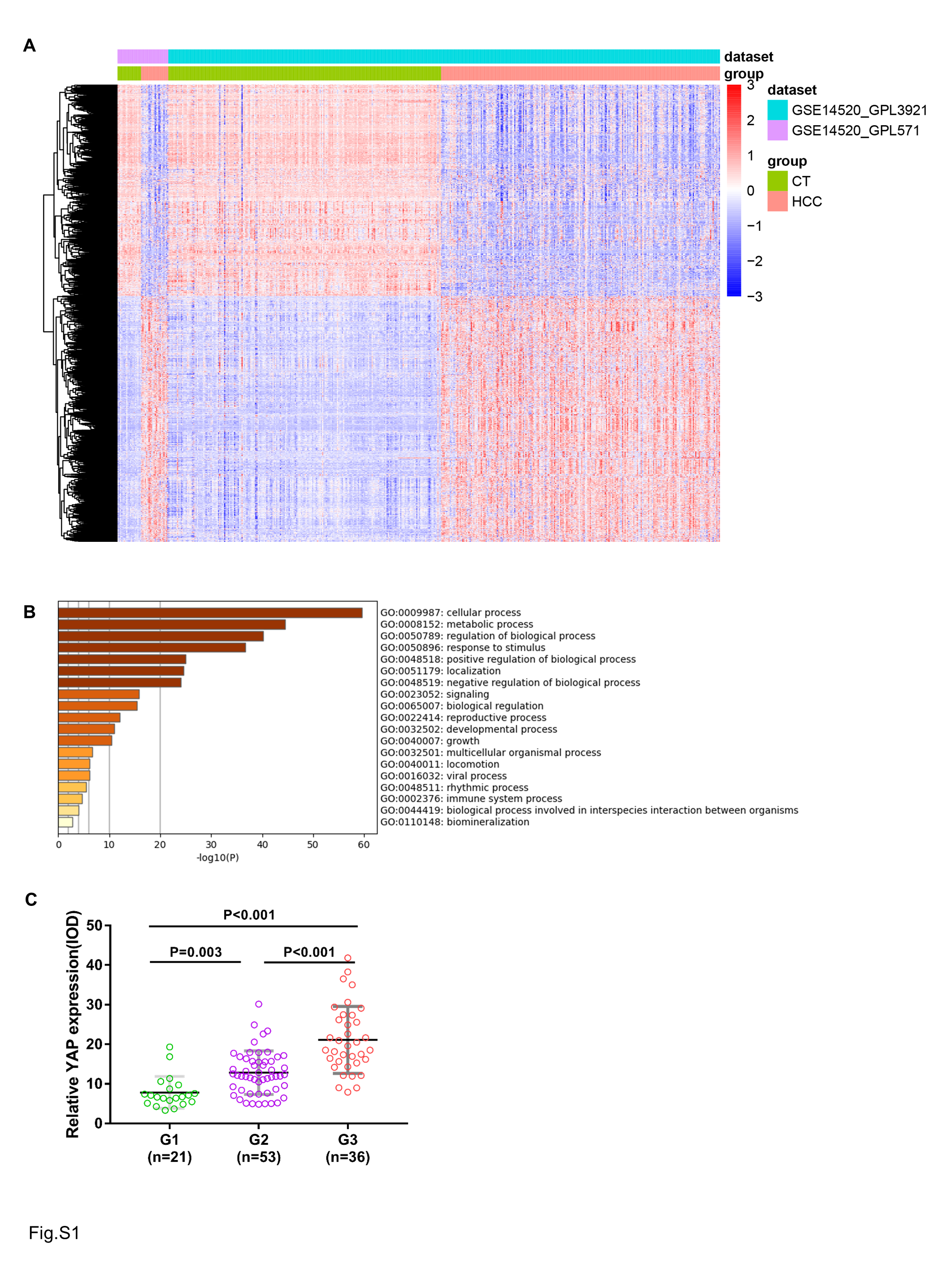

Supplement: Supplementary file 1 — Additional file 1: Figure S1. (A) Differential gene expression between liver cancer and the control tissues in the GSE14520 dataset was analyzed, with the patterns of differential gene expression (adjusted P < 0.05, ∣logFC∣≥ 0.5) being organized into a heat map. (B) Gene Ontology (GO) and Kyoto Encyclopedia of Genes and Genomes (KEGG) enrichment analysis of DEGs of tumor samples with normal ones. (C) YAP IHC staining in liver cancer patient samples. Representative YAP-stained images of Grade 1 (G1), Grade 2 (G2), and Grade 3 (G3) liver cancer tumor tissues have been quantified. [file 12967_2022_3758_MOESM1_ESM.tif]

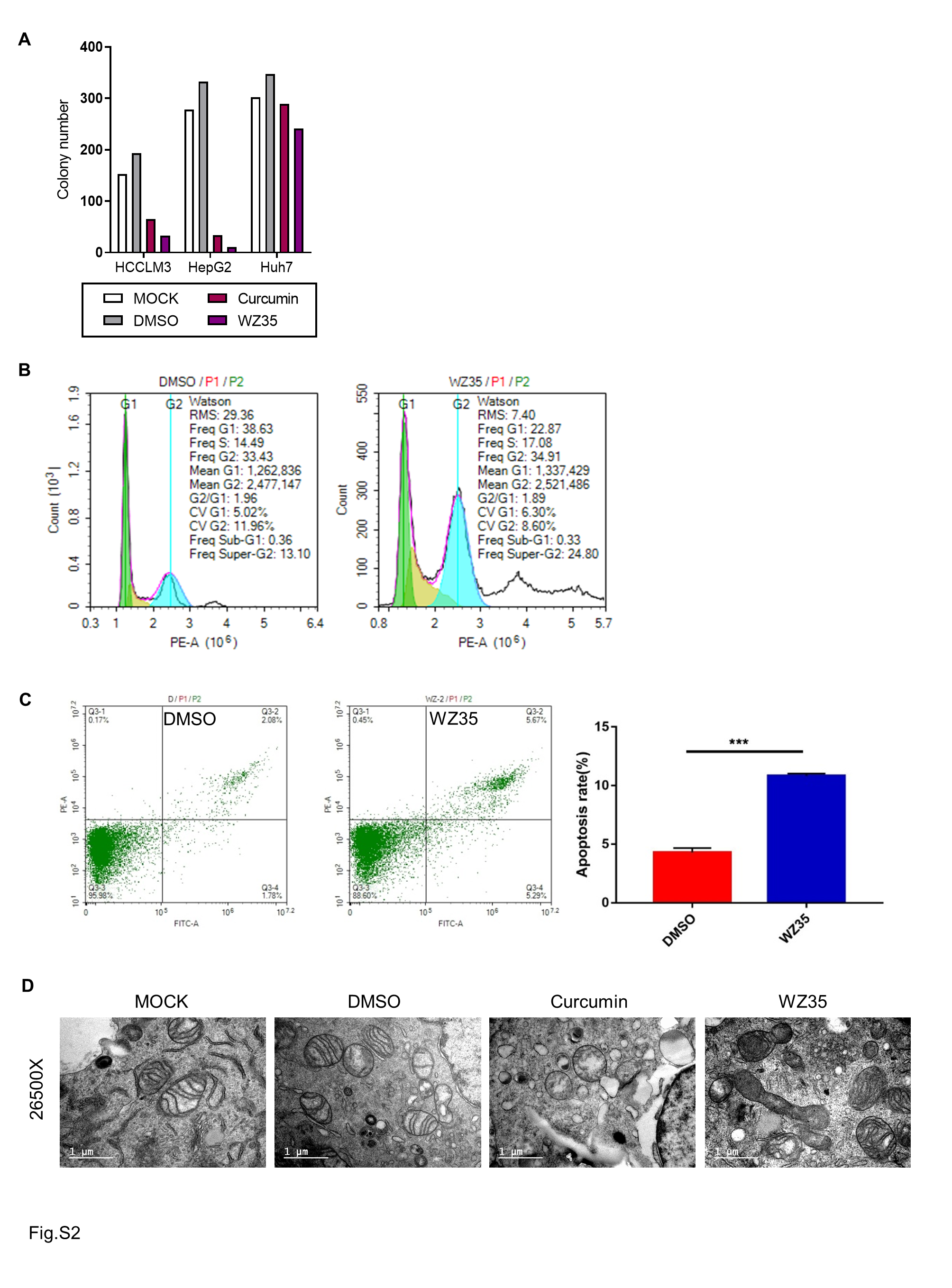

Supplement: Supplementary file 2 — Additional file 2: Figure S2. (A) Quantifications of Colony formation assays. (B) Flow cytometry analysis was utilized in the control or WZ35-treated HCCLM3 cells to measure the possible arrest in the cell cycle. Representative fitting curve of the cell cycle qualitatively illustrated the percentage of cells in G1, S and G2/M phase. (C) Apoptosis was assessed via flow cytometry in HCCLM3 cells with or without the treatment of WZ35 (10 μg/mL) for 18h using Annexin V-FITC/PI double staining, visualized by scatter plots. Percentages of apoptosis were quantified and the results have been presented as the mean ± standard error from independent experiments in triplicate. ***P < 0.001, student’s t test. (D) Transmission electron micrographic (TEM) imaging showed changes in the morphology of HCCLM3 cells subsequent to the treatment of curcumin (10 μg/mL) or WZ35 (10 μg/mL) respectively. [file 12967_2022_3758_MOESM2_ESM.tif]

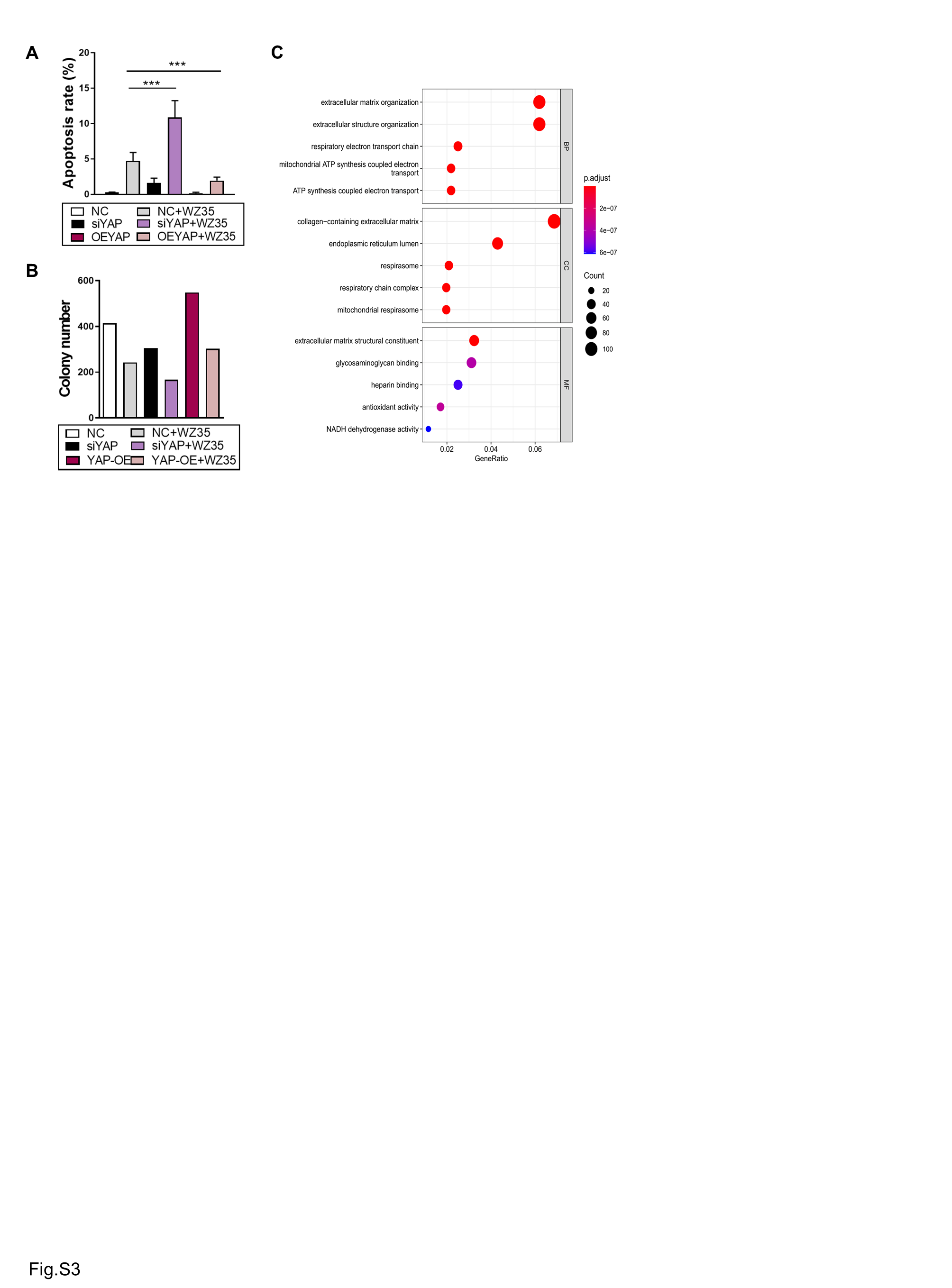

Supplement: Supplementary file 3 — Additional file 3: Figure S3. (A and B) Representative images and/or quantifications of DAPI staining assays (A) and colony formation assays (B) have been shown. All these results are presented as the mean ± standard error from independent experiments in triplicate. **P < 0.01, ***P < 0.001, student’s t test. (C ) Enriched gene ontology (GO) (C) involved by YAP expression related genes. [file 12967_2022_3758_MOESM3_ESM.tif]

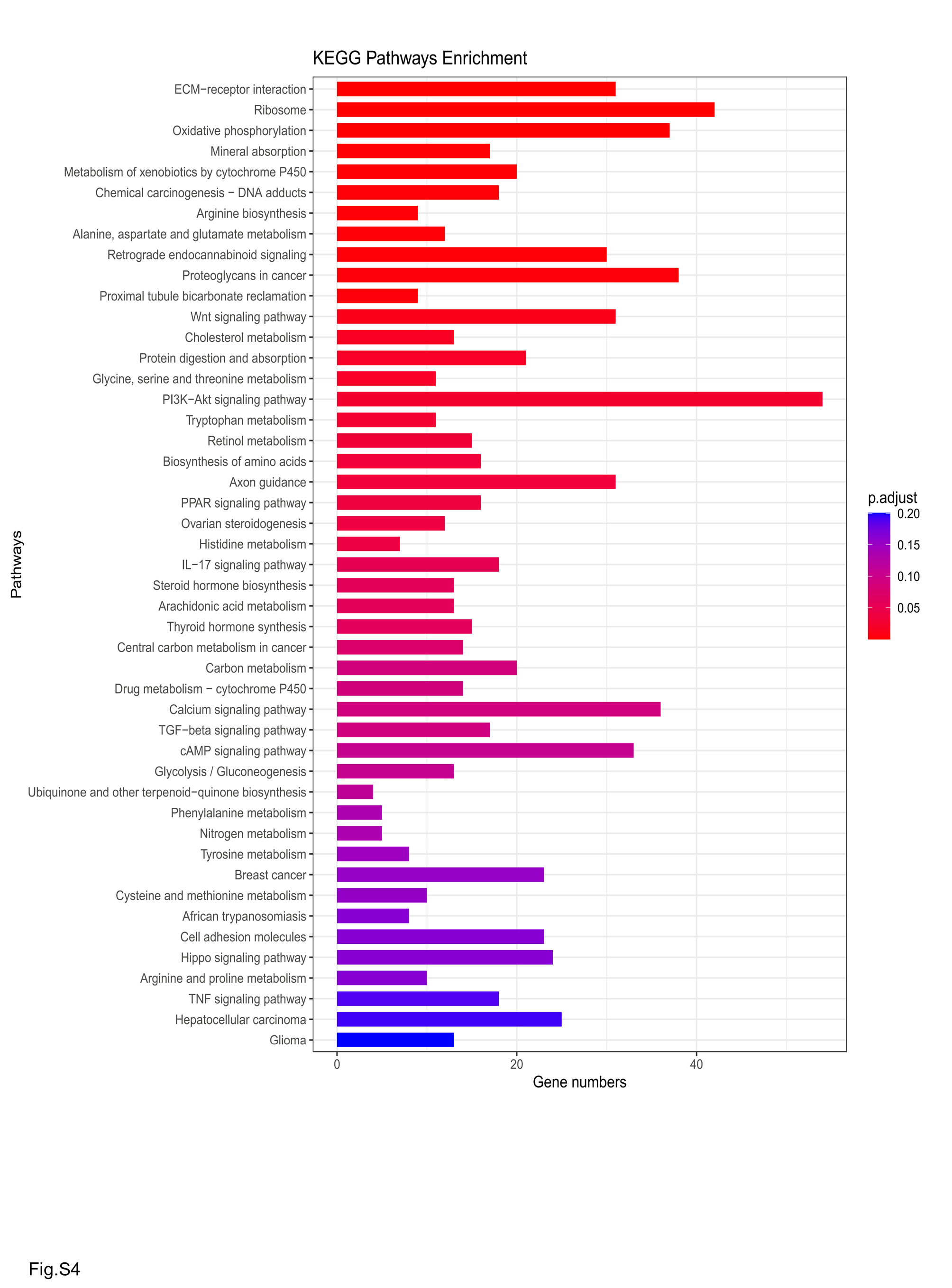

Supplement: Supplementary file 4 — Additional file 4: Figure S4. (A) KEGG pathways involved by YAP expression related genes. [file 12967_2022_3758_MOESM4_ESM.tif]

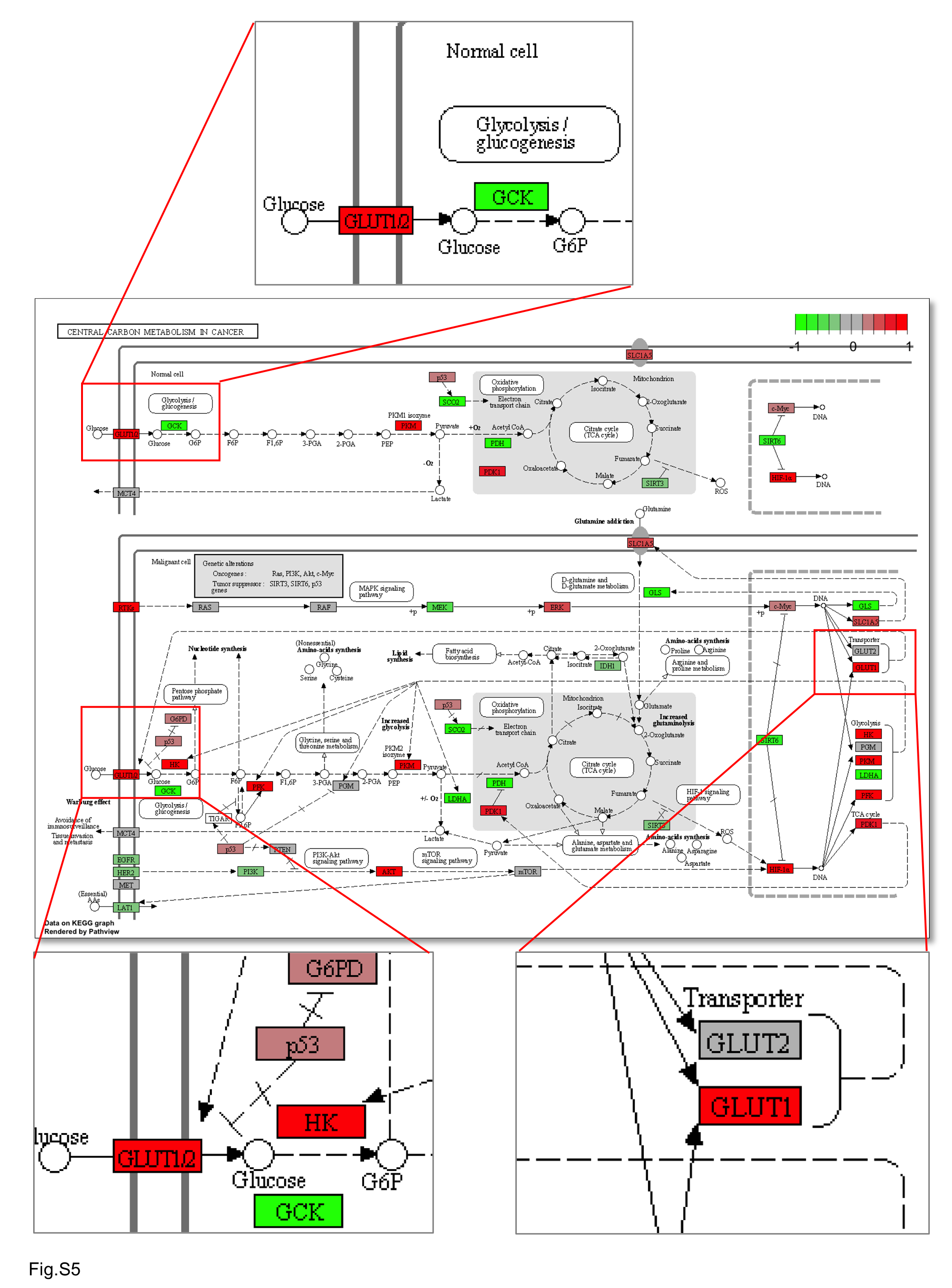

Supplement: Supplementary file 5 — Additional file 5: Figure S5. Genes in central carbon metabolism pathway of YAP high expression group on KEGG graph rendered by pathview. Gene expression levels are indicated as significantly higher (red), unchanged (gray), or lower (green). [file 12967_2022_3758_MOESM5_ESM.tif]

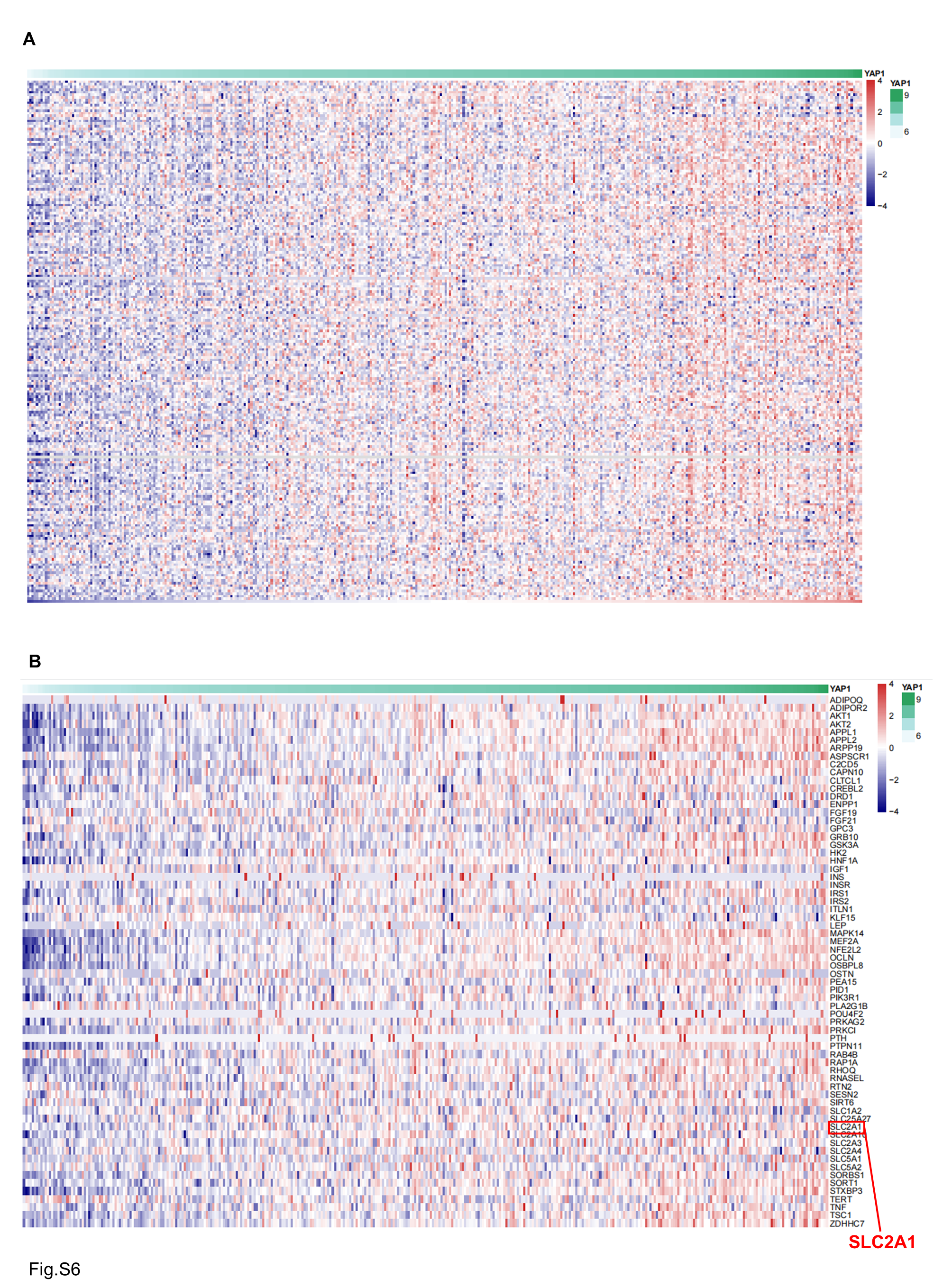

Supplement: Supplementary file 6 — Additional file 6: Figure S6. (A) Heatmap visualizing DEGs' in liver cancer samples from TCGA datasets. (B) Heatmap visualizing glucose metabolism genes' expression levels exposed to different YAP1 expression in liver cancer samples from TCGA datasets. [file 12967_2022_3758_MOESM6_ESM.tif]

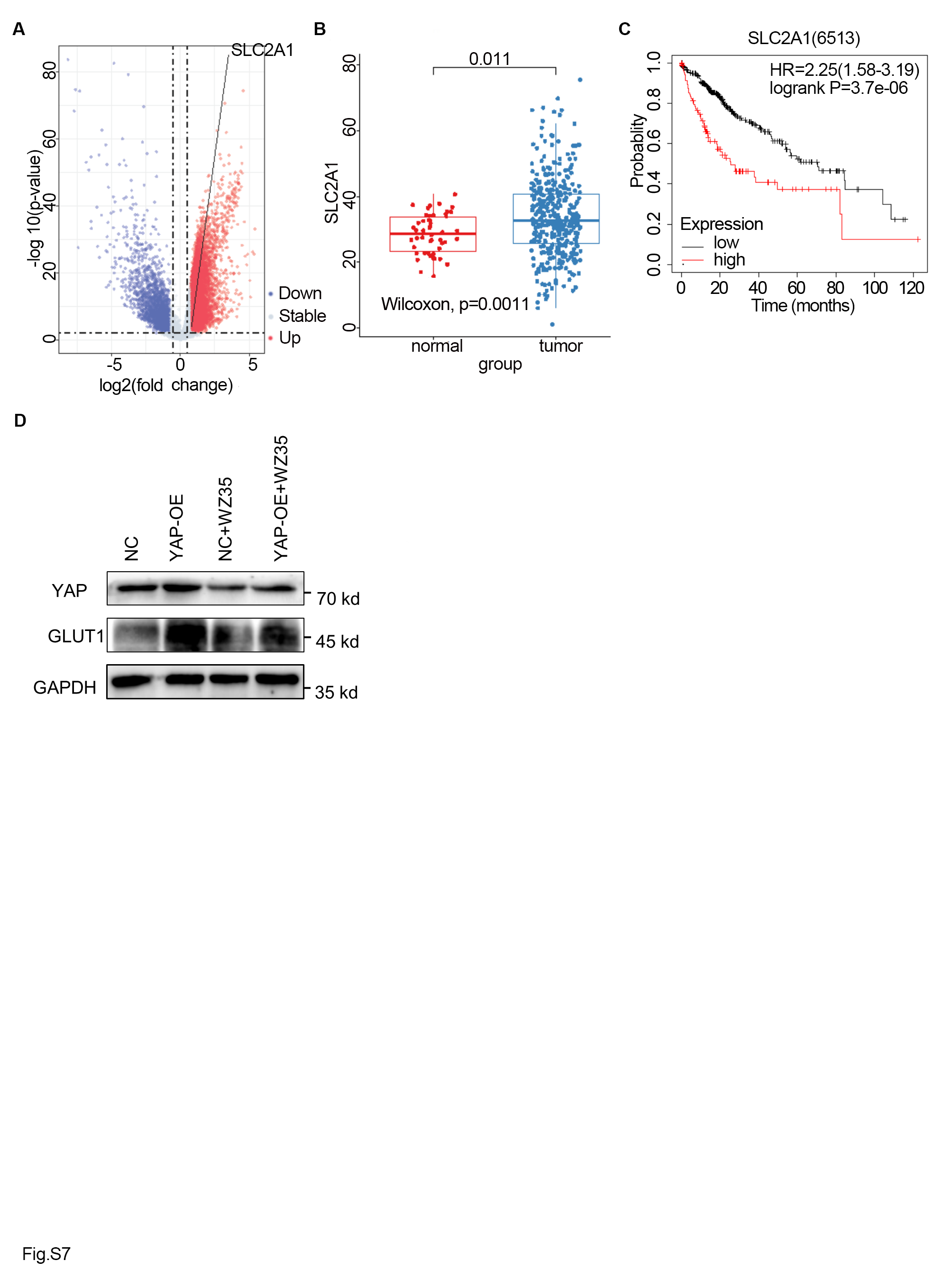

Supplement: Supplementary file 7 — Additional file 7: Figure S7. (A) Volcano plot of RNA-seq data from GEO database with SLC2A1 being marked out. (B) Box plot exhibits the distinct expression of SLC2A1 in the normal and the tumor samples, with the blue box being the representative of the tumor samples and the red box of the normal samples. (C) Kaplan–Meier analysis displaying survival for liver cancer patients stratified by expression levels of SLC2A1 (P = 3.7×10^-6^). (D) Western blotting analysis of the YAP and GLUT1 protein level of HCCLM3 cells treated with WZ35 and plasmid. [file 12967_2022_3758_MOESM7_ESM.tif]

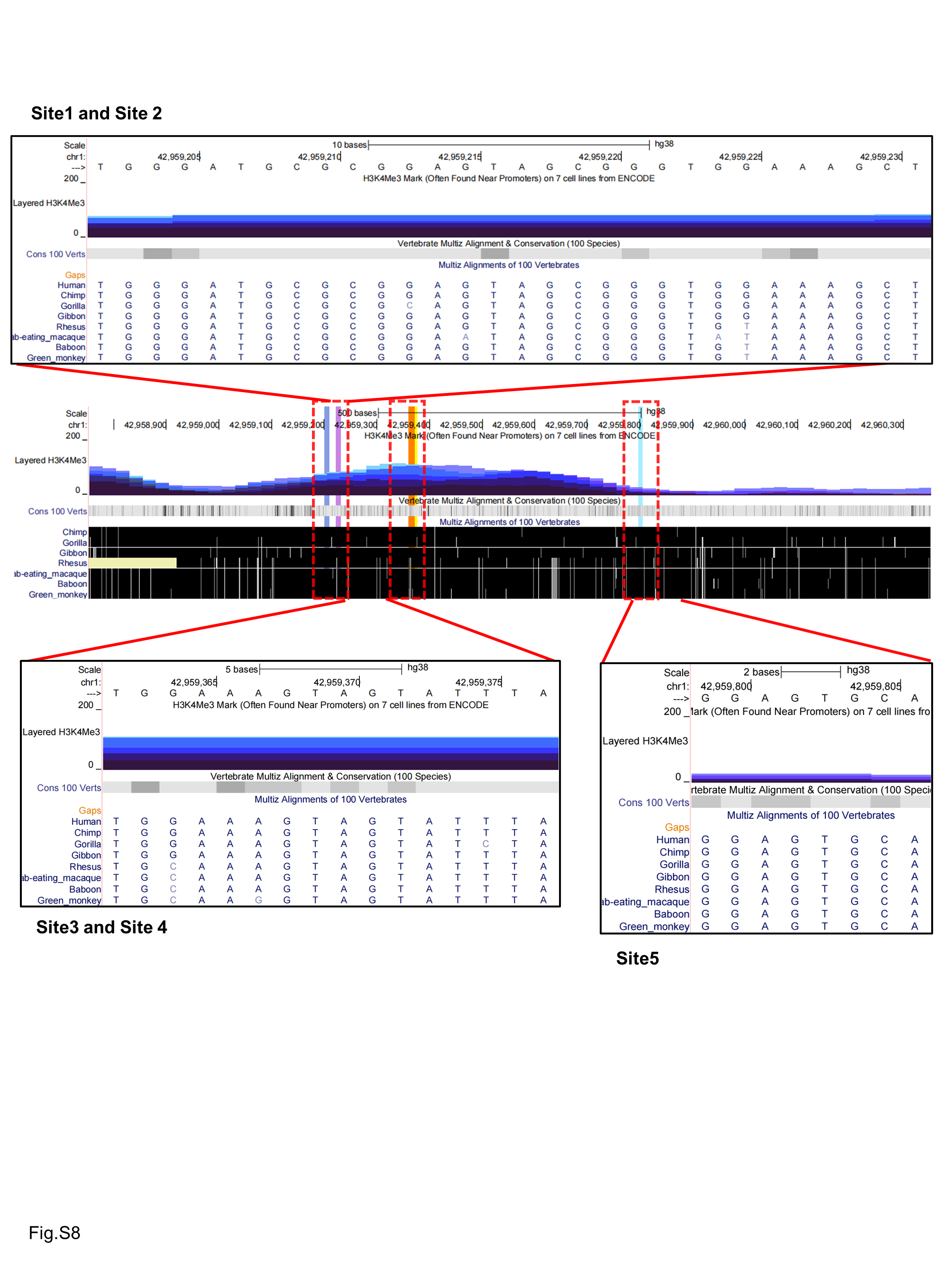

Supplement: Supplementary file 8 — Additional file 8: Figure S8. The evolutionary conservation of GLUT1 promoter and the modified state of histone methylation were depicted using the University of California, Santa Cruz (UCSC) genome browser. [file 12967_2022_3758_MOESM8_ESM.tif]

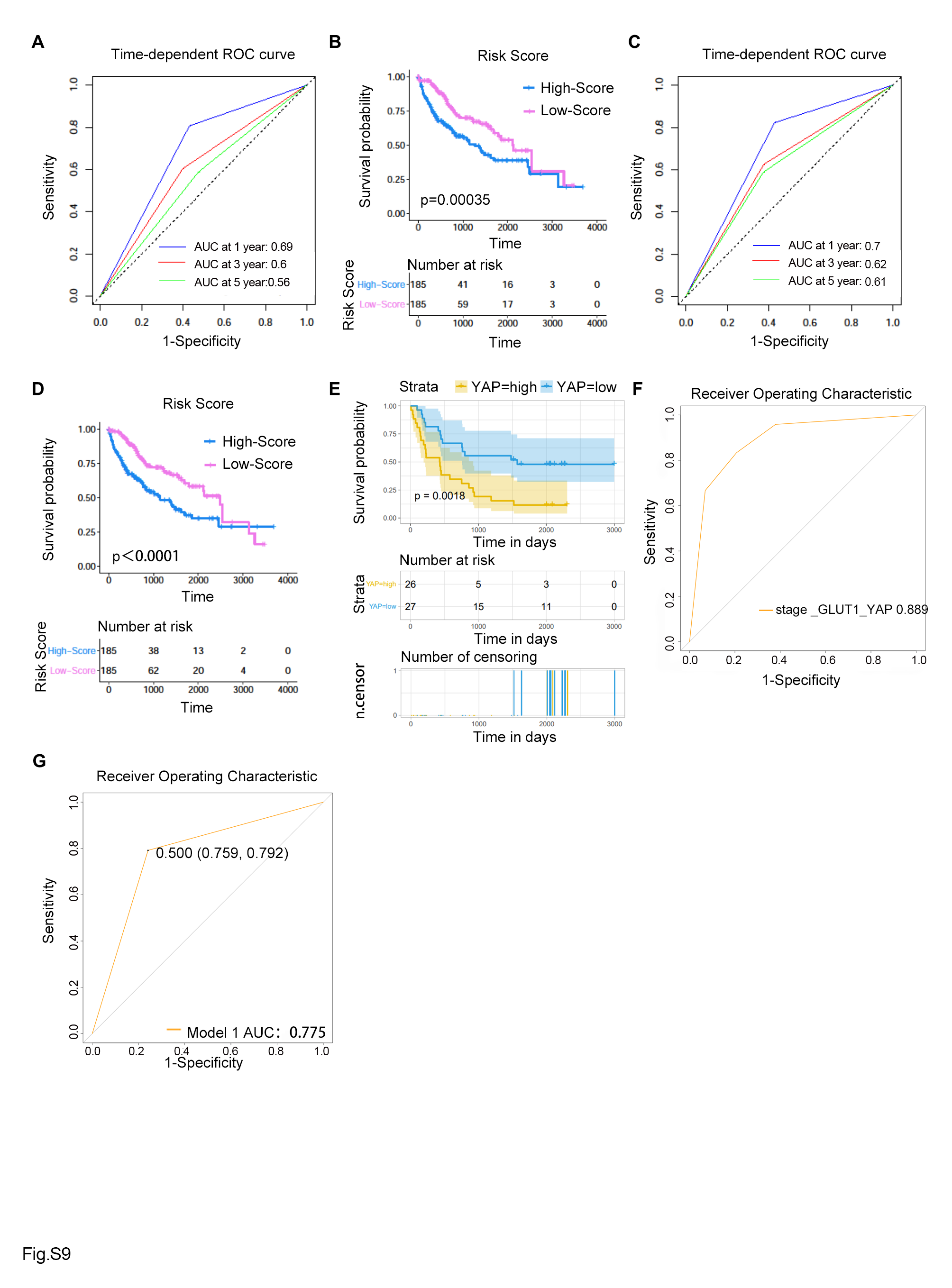

Supplement: Supplementary file 9 — Additional file 9: Figure S9. (A and C) Time-dependent ROC curves analyzing the potential value of expression levels of SLC2A1 (A) and combination of SLC2A1 and YAP1 (C) in diagnosis of liver cancer. (B and D) Kaplan–Meier analysis displaying survival rate for liver cancer samples from GEO datasets according to the expression levels of single factor of SLC2A1 (B) (P = 0.00035) and multi factors of YAP1 and SLC2A1 (D) (P < 0.0001). High integrated expression levels of YAP1 and SLC2A1 were related to the poor survival and prognosis. (E) Kaplan-Meier approach visualized the relationship between the expression of YAP and OS in liver cancer patients (n = 53, P = 0.0018). (F) ROC calibration plots of the nomogram for combined YAP and GLUT1：The AUC was 0.889. (G) ROC calibration plots of the nomogram for GLUT1: The AUC was 0.775. [file 12967_2022_3758_MOESM9_ESM.tif]
